# Body temperature variation controls pre-mRNA processing and transcription of antiviral genes and SARS-CoV-2 replication

**DOI:** 10.1093/nar/gkac513

**Published:** 2022-06-17

**Authors:** Bruna Los, Marco Preußner, Kathrin Eschke, Ricardo Martin Vidal, Azza Abdelgawad, Didrik Olofsson, Sandra Keiper, Margarida Paulo-Pedro, Alica Grindel, Stefan Meinke, Jakob Trimpert, Florian Heyd

**Affiliations:** Laboratory of RNA Biochemistry, Institute of Chemistry and Biochemistry, Freie Universität Berlin, Takustrasse 6, 14195 Berlin, Germany; Laboratory of RNA Biochemistry, Institute of Chemistry and Biochemistry, Freie Universität Berlin, Takustrasse 6, 14195 Berlin, Germany; Omiqa Bioinformatics, Altensteinstraße 40, 14195 Berlin, Germany; Omiqa Bioinformatics, Altensteinstraße 40, 14195 Berlin, Germany; Omiqa Bioinformatics, Altensteinstraße 40, 14195 Berlin, Germany; Institute of Virology, Freie Universität Berlin, Robert-von-Ostertag-Straße 7-13, 14163 Berlin, Germany; Laboratory of RNA Biochemistry, Institute of Chemistry and Biochemistry, Freie Universität Berlin, Takustrasse 6, 14195 Berlin, Germany; Laboratory of RNA Biochemistry, Institute of Chemistry and Biochemistry, Freie Universität Berlin, Takustrasse 6, 14195 Berlin, Germany; Laboratory of RNA Biochemistry, Institute of Chemistry and Biochemistry, Freie Universität Berlin, Takustrasse 6, 14195 Berlin, Germany; Laboratory of RNA Biochemistry, Institute of Chemistry and Biochemistry, Freie Universität Berlin, Takustrasse 6, 14195 Berlin, Germany; Omiqa Bioinformatics, Altensteinstraße 40, 14195 Berlin, Germany; Laboratory of RNA Biochemistry, Institute of Chemistry and Biochemistry, Freie Universität Berlin, Takustrasse 6, 14195 Berlin, Germany

## Abstract

Antiviral innate immunity represents the first defense against invading viruses and is key to control viral infections, including SARS-CoV-2. Body temperature is an omnipresent variable but was neglected when addressing host defense mechanisms and susceptibility to SARS-CoV-2 infection. Here, we show that increasing temperature in a 1.5°C window, between 36.5 and 38°C, strongly increases the expression of genes in two branches of antiviral immunity, nitric oxide production and type I interferon response. We show that alternative splicing coupled to nonsense-mediated decay decreases STAT2 expression in colder conditions and suggest that increased STAT2 expression at elevated temperature induces the expression of diverse antiviral genes and SARS-CoV-2 restriction factors. This cascade is activated in a remarkably narrow temperature range below febrile temperature, which reflects individual, circadian and age-dependent variation. We suggest that decreased body temperature with aging contributes to reduced expression of antiviral genes in older individuals. Using cell culture and *in vivo* models, we show that higher body temperature correlates with reduced SARS-CoV-2 replication, which may affect the different vulnerability of children versus seniors toward severe SARS-CoV-2 infection. Altogether, our data connect body temperature and pre-mRNA processing to provide new mechanistic insight into the regulation of antiviral innate immunity.

## INTRODUCTION

Endothermic organisms have evolved an elaborate thermoregulatory system to maintain their body temperature in a narrow temperature range. In humans, neurons in the preoptic area of the hypothalamus act as a thermostat that controls core body temperature through inducing, for example, behavioral adaptation, heat dissipation and conservation or thermogenesis (1). Thermal homeostasis results in core body temperature mostly between 36.5 and 37.5°C in healthy humans (2). Human core body temperature is largely independent of the ambient temperature, but shows subtle changes depending, for example, on endogenous rhythms or behavioral patterns (3). Rhythmic changes in core body temperature are observed daily with the circadian clock but also monthly with the female hormone cycle (4,5). Behavioral patterns that alter core body temperature are physical exercise, which increases core body temperature, or fasting, which can have the opposite effect. In addition, young children display an average core body temperature that is higher than that in adults and older individuals (6). However, all these changes are subtle, in most cases within a range of 1°C between 36.5 and 37.5°C, e.g. in a circadian cycle or associated with aging. Daily changes in ambient temperature and also the temperature range typically used in cell culture and *in vitro* experiments when addressing changes in gene expression or enzymatic activity by far exceed this 1°C differential of core body temperature. It thus remains an open question whether and how variation in the narrow range of healthy human core body temperature contributes to controlling gene expression programs and cellular functionality.

Earlier work has uncovered a family of receptors, TRP channels, which react to changes in temperature (7). Different members of the TRP channels respond to different temperature ranges, mostly with respect to changes in the ambient temperature, and their function is therefore usually studied in skin and primary sensory neurons (8). We have recently described a second class of thermo-sensors, a family of kinases called CLKs, which are broadly expressed and control the phosphorylation state of SR proteins (9). We have shown that the activation segment of CLKs undergoes temperature-controlled structural rearrangements to control kinase activity precisely in the narrow body temperature range below fever, for mice and humans between 36 and 38°C, thus translating body temperature changes into kinase activity and SR protein phosphorylation and ultimately global changes in alternative splicing and gene expression (4,9). While this work has started to elucidate structural and molecular mechanistic details, functional implications of body temperature-controlled alternative splicing and gene expression remain largely speculative.

In addition to physiological changes in core body temperature, pathological changes occur, most notably during an infection with fever. Fever is a condition of elevated core body temperature often defined as temperature above 38°C (10). It is a reaction to invading pathogens that are recognized by a variety of receptors eventually leading to the production and secretion of pyrogenic cytokines that cause the hypothalamus to increase the temperature set point. Fever has been suggested to act in several ways to clear pathogens, e.g. by reducing the replication of pathogens while increasing proliferation, mobility and phagocytic activity of host immune cells (11). Accordingly, reducing or preventing fever during an infection increases mortality in diverse model systems (11). Above 39°C, fever also induces the heat shock response, which, e.g. by activating heat shock factor-1, contributes to control the expression of chemokines and cytokines and thus plays an important role in orchestrating an immune response (12). Most studies that have analyzed molecular mechanisms controlling fever or the heat shock response have used temperatures of 39°C or above, thus leaving the sub-pathological temperature range unexplored. For example, several studies have addressed the role of increased body temperature in viral infection and replication, but mostly focused on 39°C, a condition that corresponds to high fever (13–16).

Since the beginning of the SARS-CoV-2 pandemic in early 2020, countless studies have been published, ranging in scope from basic molecular and structural biology to the development of therapeutic and vaccination strategies. As for other viruses, the innate immune response represents the first line of defense against invading SARS-CoV-2 and is key to control the early phase of infection (17,18). A more efficient type I interferon response in the early phase likely contributes to a milder course of the disease, while a failure to efficiently control the virus in the early phase favors a more severe outcome (19,20). Individual variation in the efficiency of the innate immune response immediately following SARS-CoV-2 infection thus likely represents an important factor determining disease outcome. Despite the obvious relevance for individual and public health, variables that determine the precise activity of the antiviral innate immunity remain largely unknown (17). Body temperature is an omnipresent variable that has so far been neglected when analyzing the first encounter of SARS-CoV-2 with the host. Here, we show that alterations in the sub-pathological range of core body temperature, between 36.5 and 37.5°C, control the expression of antiviral genes. Increased temperature alters STAT2 pre-mRNA processing to induce the type I interferon response and nitric oxide (NO) production, thus activating two antiviral strategies. We furthermore show that many SARS-CoV-2 restriction factors are expressed to a higher level at higher temperature, that their expression is reduced with aging and that higher temperature at the time point of infection reduces SARS-CoV-2 replication in the cell culture model with a similar trend observed *in vivo*. Together, we present a new regulatory layer of antiviral innate immunity, which is controlled by subtle variation in the sub-pathological body temperature range.

## MATERIALS AND METHODS

### Cell culture

RAW 264.7, HeLa, Caco-2, Calu-3, 3T3 and N2a cells were maintained in DMEM High Glucose (Biowest), supplemented with 10% (v/v) fetal bovine serum (FBS) and 1% (v/v) penicillin/streptomycin (Invitrogen). Jurkat cells were maintained in RPMI 1640 (Biowest) supplemented with 10% (v/v) FBS and 1% (v/v) penicillin/streptomycin. The cells are tested for mycoplasma contamination monthly using a PCR-based assay.

Bone marrow-derived primary macrophages were isolated from C57BL/6 wild-type mice using fluorescence-activated cell sorting (BD FACSMelody™). Briefly, bone marrow cells were pipetted through a 70-μm cell strainer, centrifuged at 500 × *g* and 4°C for 5 min, lysed in 1 ml of red blood cells lysis buffer and incubated for 10 min at room temperature. Cells were then centrifuged at 500 × *g* and 4°C for 5 min and resuspended in 100 μl of 1× phosphate-buffered saline (PBS). The following antibodies were used for reverse staining: CD4-FITC (Miltenyi, 1:20), CD8-FITC (Miltenyi, 1:20) and CD3-FITC (Miltenyi, 1:20). Sorted cells were seeded in 12-well plates (0.5 × 10^6^ cells per well) and maintained in DMEM medium supplemented with 10% (v/v) FBS, 1% (v/v) penicillin/streptomycin and l-glutamine.

For temperature experiments, cells were seeded and allowed to adhere at 37°C. The cells were subsequently shifted to incubators set to 34, 35, 36.5, 37, 37.5, 38 or 39°C as indicated and incubated at the respective temperature for 12 h unless otherwise stated.

Inhibitors were used at the following concentrations: actinomycin D (ActD; Sigma, 5 μg/ml), cycloheximide (CHX; Carl Roth, 40 μg/ml), TG003 (Cayman Chemical Company, 50 μM) and ruxolitinib (10 μM). Dimethyl sulfoxide (DMSO) was used as a control.

Cells were also stimulated with 0.1 μg/ml of LPS (eBioscience™ Lipopolysaccharide Solution 500×, Invitrogen, #00-4976), 1 ng/ml of phorbol myristate acetate (PMA, Sigma-Aldrich) and 100 U/ml of Universal Type I Interferon (IFN, R&D Systems, #11200-1).

### Antisense oligonucleotide transfection

N2a and 3T3 cells were transfected in six-well plates with 200 μM/ml of 2′-MOE phosphorothioate antisense oligonucleotides (ASOs, Microsynth) flanking the alternative 5′ splice site in exon 11 of Stat2 using Lipofectamine 2000 (Invitrogen) following the manufacturer’s protocol. Forty-eight hours post-transfection, the RNA was extracted and RT-PCR and RT-qPCR were performed as described below.

ASO sequences: ASO1: 5′-TGGGTACCTGTGCTTCTA-3′; ASO2: 5′-TGAAGTGGGTACCTGTGC-3′.

### Generation of CRISPR/Cas9-edited N2a cells

For CRISPR/Cas9-mediated deletion of the alternative 5′ splice site in exon 11 of Stat2, sgRNA was designed using the Benchling tool and cloned into the px458 plasmid. N2a cells were transfected in six-well plates using Lipofectamine 2000 (Invitrogen) following the manufacturer’s protocol. Forty-eight hours post-transfection, GFP-positive single cells were sorted using the BD FACSMelody™. DNA and RNA from expanded clones were extracted and analyzed by PCR. sgRNA sequences (5′–3′): sgRNA1: taaaacagccaacaggcac; sgRNA3: caggtacccacttcacggc; sgRNA4: aggctagtttcattgtcc.

### RNA-seq

For RNA-seq, RAW 264.7 cells were seeded on 10-cm dishes and grown for ∼48 h at 37°C to ∼75% confluence. Cells were then shifted to 34, 37 and 38°C, and incubated for 12 h. Total RNA was extracted as described below. Sequencing libraries were prepared in triplicates (for 37°C, *n* = 4) from total RNA by poly(A) selection using the TruSeq mRNA Library Preparation Kit. Sequencing was performed on an Illumina HiSeq 2500 system with V4 sequencing chemistry, generating around 50 million 150-bp paired-end reads per sample. We performed differential gene expression (DGE) analysis by first mapping reads to the mouse genome (GRCm38) with gene annotation (GENCODE M21) using star (version 2.7.5b) on default settings. Gene counting was then performed with featureCounts (version 1.6.4) and the resulting count matrix was used as input for DESeq2 (version 1.28.1). DGE analysis was performed by running DESeq2 with fitType = ‘local’. Log_2_ fold change shrinkage was performed by the adaptive shrinkage algorithm to reduce the effect of low expression levels on fold changes. Significantly differential genes were identified by filtering on adjusted *P*-value ≤0.001 and absolute log_2_ fold change ≥0.8. For heat maps, mean normalized expression counts were calculated for each condition and normalized to the sum of all three temperatures. For GO term enrichment, we used the PANTHER GO enrichment analysis for biological processes (http://geneontology.org/) comparing changed genes with all expressed genes in RAW 264.7 cells. Only GO enrichments with a false discovery rate <1 × 10^–5^ are presented.

### RNA extraction, RT-PCR and RT-qPCR

Total RNA was extracted using RNATri (Bio&Sell) as described in the user manual, followed by DNase digest to remove DNA contamination. For chromatin-associated RNA extraction, cells were resuspended in 100 μl of cold CTX buffer (10 mM HEPES–NaOH, pH 7.9, 1.5 mM MgCl_2_, 10 mM KCl) and incubated on ice for 5 min. Furthermore, 100 μl of cold CTX buffer supplemented with 0.2% (v/v) NP-40 was added to the samples, followed by incubation on ice for 5 min. Nuclei were pelleted by centrifugation at 4100 × *g* for 3 min at 4°C. The nuclear fraction was resuspended in 40 μl of NX buffer (20 mM HEPES–NaOH, pH 7.9, 1.5 mM MgCl_2_, 420 mM KCl, 0.2 mM EDTA, 25% (v/v) glycerol). The chromatin-associated RNA was then isolated using RNATri (Bio&Sell).

RT-PCRs were done as previously described (4). Briefly, low-cycle PCR with a [^32^P]-labeled forward primer was performed, and products were separated by denaturing PAGE and quantified using a phosphorimager (Typhoon 9200, GE Healthcare) and ImageQuantTL software. Quantifications are given as mean values of several technical and biological replicates, error bars represent standard deviation and *P*-values were calculated using Student’s unpaired *t*-test. Significance is indicated by asterisks (*P < 0.05; **P < 0.01; ***P < 0.001; ^****^P < 0.0001; ns = not significant.).

For RT-qPCR, up to six gene-specific primers were combined in one RT reaction using 1 μg of purified RNA. qPCR was then performed in a 96-well format using the Biozym Blue S’Green qPCR Kit and a Bioer LineGene 9600 Plus instrument. qPCRs were performed in duplicates, mean values were used to normalize expression to the mRNA of *Hprt* (mouse cell lines) or *GAPDH* (human cell lines) (ΔCT) and Δ(ΔCT)s were calculated for different conditions. Quantifications are given as mean values, error bars represent standard deviation and *P*-values were calculated using Student’s unpaired *t*-test. Significance is indicated by asterisks (*P < 0.05; **P < 0.01; ***P < 0.001; ^****^P < 0.0001; ns = not significant.). See Supplementary Table S2 for primer sequences.

### Western blot

Whole-cell lysates and nuclear/cytoplasmic fractionations were prepared as previously described (21). Equal amounts of denatured protein were separated on 10% SDS-PAGE and transferred onto a nitrocellulose membrane. The membrane was incubated in 2% BSA–LS TBST or 5% non-fat dry milk–LS TBST at room temperature for 1 h and subsequently with primary antibody overnight at 4°C. The following primary antibodies were used: rabbit anti-phospho-Stat2 (Tyr690), 1:1000 (#4441, Cell Signaling Technology); rabbit anti-phospho-Stat3 (Tyr705), 1:1000 (#9131, Cell Signaling Technology); mouse anti-RIG-I, 1:1000 (sc-376845, Santa Cruz Biotechnology); rabbit anti-Stat2 (mouse specific), 1:1000 (#4597, Cell Signaling Technology). Antibody recognition was detected with the secondary antibody linked to horseradish peroxidase (anti-mouse IgG HRP, 1:5000, #7076s, Cell Signaling Technology; anti-rabbit IgG HRP, 1:5000, #7074s, Cell Signaling Technology) at room temperature for 1 h. hnRNP-L bands (anti-hnRNP-L, Santa Cruz Biotechnology, #sc-32317, 1:2000) were used as a loading control. The immunoreactive bands were visualized with Pierce™ ECL Western Blotting Substrate (Thermo Fisher Scientific) using the Amersham Imager 600 (GE Healthcare). The bands were quantified using ImageQuantTL software.

### Nitric oxide assay

Cells were incubated with 1.25 or 2.25 μg/ml of LPS at 37 or 39°C for 16 h to induce endogenous NO formation. For measuring intracellular NO levels, the Nitric Oxide Assay Kit (flow cytometry—orange, #ab219933) was used according to the manufacturer’s protocol. The analyses were carried out with the BD FACSMelody™.

### SARS-CoV infection and quantification

SARS-CoV-2 virus stocks were prepared from passage 3 of an early 2020 SARS-CoV-2 B.1 outbreak isolate (BetaCoV/Germany/BavPat1/2020) on Vero E6 cells. All work related to authentic SARS-CoV-2 was performed under appropriate BSL-3 safety conditions (Institut für Virologie, Freie Universität Berlin). Virus was propagated on Calu-3 (ATCC HTB-55) and Caco-2 (ATCC HTB-37) cells. Titrations were performed on Vero E6 cells (ATCC CRL-1586) in minimal essential medium (PAN Biotech, Aidenbach, Germany) supplemented with 10% FBS (PAN Biotech, Aidenbach, Germany), 100 IU/ml penicillin G and 100 μg/ml streptomycin (Carl Roth, Karlsruhe, Germany).

For temperature-controlled SARS-CoV-2 infection experiments, Calu-3 and Caco-2 cells were grown to 80% confluence on six-well plates (Sarstedt, Nuembrecht, Germany) and maintained at 37 or 39°C for 16 h prior to infection. SARS-CoV-2 infection was performed by adding virus stock at an MOI of 0.01 to cell monolayers and subsequent incubation at 37°C for 90 min. Virus inoculum was then removed, cells were washed with PBS and fresh cell culture medium was added before cells were returned to 37°C and harvested at 24 and 48 h post-infection. All cell culture experiments were performed in 12 biological replicates for each cell line and condition.

To determine virus titers as plaque forming units, Vero E6 cells grown in 12-well plates were infected with 100 μl of serial 10-fold dilutions of virus. After 75 min of incubation, the viral inoculum was removed and cells were overlaid with EMEM 2× containing 1.5% microcrystalline cellulose and carboxymethyl cellulose sodium (Vivapur MCG, JRS Pharma). Forty-eight hours after infection, cells were fixed with 4% formalin. The wells were stained with 1 ml of a 0.75% aqueous methylene blue solution, washed with PBS to remove residual stain and plaques were counted in an appropriate dilution to calculate virus titers in the inoculum.

## RESULTS

### A subtle temperature increase strongly increases the expression of antiviral response genes

**Figure 1.**
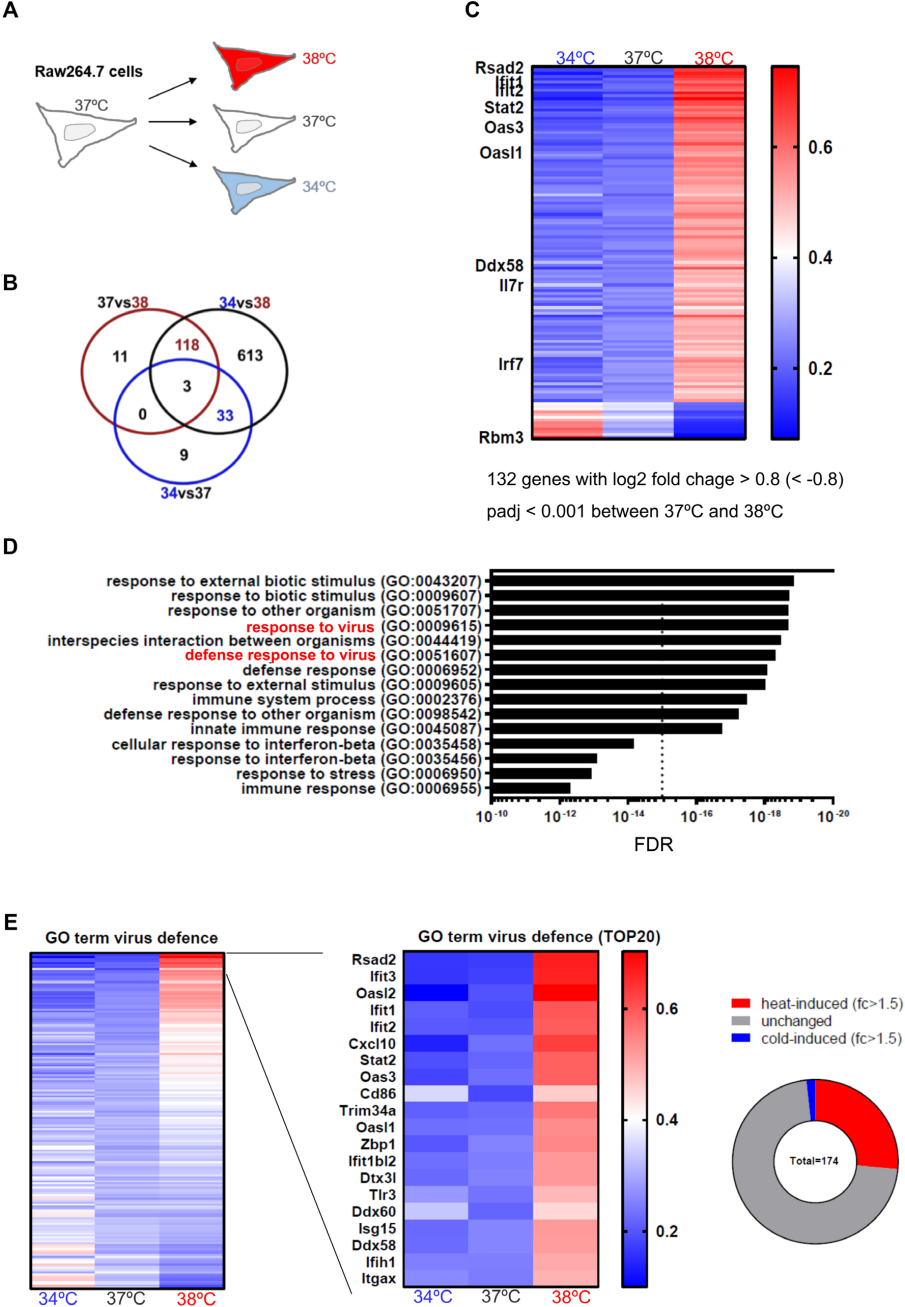
Antiviral immunity is upregulated at slightly elevated temperature in macrophages. **(A)** Experimental design. RAW 264.7 macrophages were cultured at 37°C and then shifted for 12 h to the indicated temperature. RNA-seq was then performed for three independent samples for each temperature. **(B)** Temperature-dependent changes in gene expression. Genes were considered significantly changed with a log_2_ fold change >0.8 (<−0.8) and *P*_adj_ < 0.001 between two temperatures. The Venn diagram compares significantly changed genes between 37 and 38°C (red), 34 and 37°C (blue), and 34 and 38°C (black). Note that different genes are affected by warm and cold temperatures, and that the 1°C increase from 37 to 38°C affects more genes than the 3°C decrease from 37 to 34°C. **(C)** Heat map showing significantly altered genes comparing 37 and 38°C (*n* = 132, sorted by fold change between 37 and 38°C). Each line represents one gene, cold-induced Rbm3, and heat-induced genes associated with viral defense are highlighted on the left. Note that most of the 38°C-induced genes are not affected between 34 and 37°C and/or changes do not pass our criterion for significance. **(D)** The strongest GO term enrichments of 38°C-induced genes. GO terms containing the term ‘virus’ are highlighted in red; most other GO terms are also associated with antiviral defense or innate immunity. Genes expressed in RAW 264.7 cells served as background. No GO term enrichment is found when analyzing genes induced at 34°C compared to 37°C. **(E)** Heat map as in panel (C) showing all expressed genes associated with the GO term ‘defense response to virus’ (left, *n* = 174, sorted by change from 37 to 38°C). The 20 strongest warm-induced genes are shown in the middle. See also Supplementary Table S1. Right: Over 25% of genes associated with antiviral defense are induced at warmer temperature (FC 37°C/38°C > 1.5), while only three genes are significantly induced at 34°C (FC 34°C/37°C > 1.5).

To systematically evaluate the impact of body temperature on the expression of genes related to the innate immune system, we performed RNA-seq using the mouse macrophage cell line RAW 264.7. Cells were incubated for 12 h at 34, 37 or 38°C (Figure 1A) and RNA-seq was performed in independent triplicates per condition; we obtained around 50 × 10^6^ 150PE reads in each individual sample. We used DESeq to compare changes in gene expression between the three temperatures using a fold change of >1.74 (log_2_ fold change >0.8) and *P* < 0.001 as cutoffs. While only 45 genes changed between 34 and 37°C, we found 132 changes in gene expression when increasing the temperature from 37 to 38°C (Figure 1B and Supplementary Table S1). There was only little overlap between the changes in gene expression when shifting cells from normothermia (37°C) to either hypothermia (34°C) or slightly increased temperature (38°C), suggesting a specific response to the respective temperature (Figure 1B and C). The strongest inverse correlation between temperature and gene expression (lower expression at higher temperature) was observed for the RNA binding protein Rbm3, which is known to be cold induced, showing a 3-fold decrease from 38 to 37°C and an almost 10-fold reduction between 38 and 34°C (Supplementary Figure S1A). Temperature-dependent Rbm3 expression confirms that the temperature treatment, also for the small change between 37 and 38°C, was effective. Remarkably, when comparing genes with altered expression between 37 and 38°C, the majority of genes showed increased expression at the higher temperature and the strongest changes were observed for genes related to innate immunity, interferon response and antiviral response (Figure 1C). These genes did not show a change in expression between 34 and 37°C, suggesting a specific activation of innate immunity and antiviral pathways at elevated temperature (Figure 1C). This notion is further supported by GO term analysis of genes that increase between 37 and 38°C, showing a strong and highly significant enrichment of processes such as ‘response to biotic stimuli’, ‘response to virus’ or ‘response to interferon’ among other innate immunity-related GO terms (Figure 1D). The same GO terms were enriched when comparing genes changed between 34 and 38°C, whereas genes that are differentially expressed between 34 and 37°C showed no such enrichment (Supplementary Figure S1B–D), again demonstrating that the effect is specific for elevated body temperature. Furthermore, among the 174 genes expressed in RAW 264.7 cells that are associated with the GO term ‘response to virus’, we found >25% to be induced between 37 and 38°C, whereas only very few genes showed higher expression at 34°C (Figure 1E). Taken together, these data indicate antiviral priming of macrophages specifically by a 1°C increase in temperature from 37 to 38°C, which represents the normothermic range at the transition to mild fever.

### Increased JAK-STAT signaling induces antiviral response genes at elevated temperature in the normothermic range

**Figure 2.**
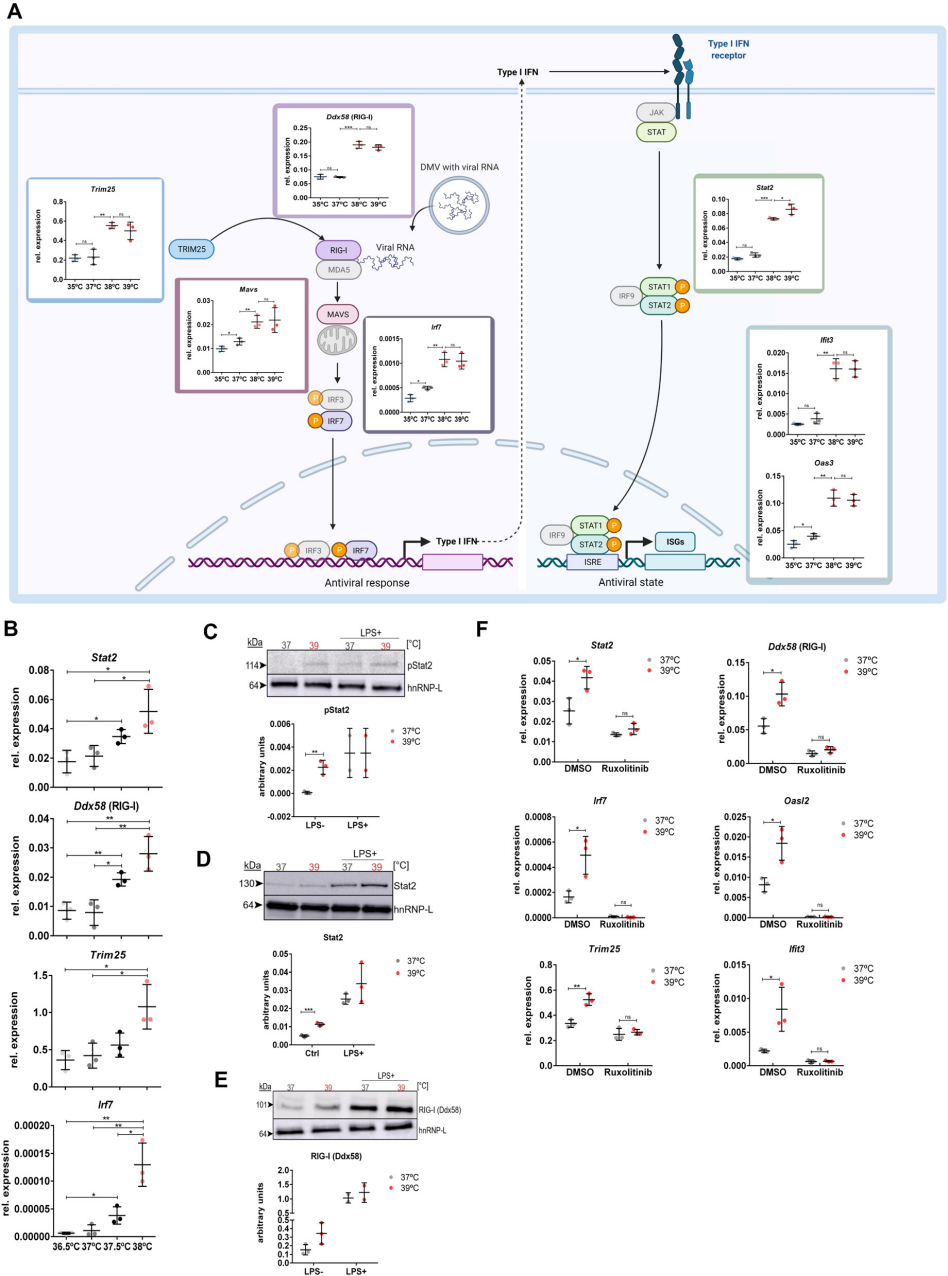
JAK-STAT signaling is required for the temperature-controlled increase of antiviral genes. **(A)** The canonical antiviral cascade, also required for defense against SARS-CoV-2, leading to the type I interferon response is shown (created with BioRender.com). Components at all steps of this pathway are activated at 38°C; the expression of all genes shown in color was increased in the RNA-seq experiment in Figure 1 (genes shown in gray were not changed). For RT-qPCR validation, RAW 264.7 cells were incubated at the indicated temperature for 12 h and gene expression was analyzed (*n* = 3, mean ± SD). All genes with a predicted increase in expression at 38°C in the RNA-seq data were confirmed by RT-qPCR (validation rate 100%). **(B)** RAW 264.7 cells were incubated at the indicated temperature for 12 h and gene expression was analyzed by RT-qPCR. mRNA expression is relative to Hprt (*n* = 3, mean ± SD). **(C)** Elevated temperature is sufficient to induce JAK-STAT signaling. RAW 264.7 cells were incubated at the indicated temperature for 12 h and phosphorylation (activation) of STAT2 was analyzed by western blot (*n* = 3, mean ± SD). As positive control, cells were incubated for 16 h with LPS (2.25 μg/ml) (*n* = 2, mean ± SD). A representative blot and quantification of three independent experiments are shown. hnRNP-L served as a loading control. **(D)** Western blot as in panel (C) showing increased expression of Stat2 also at the protein level. A representative blot and quantification relative to hnRNP-L are shown (*n* = 3). **(E)** Western blot as in panel (B) showing increased expression of RIG-I (Ddx58) also at the protein level. A representative blot and quantification relative to hnRNP-L are shown. **(F)** Inhibition of JAK-STAT signaling abolishes temperature-induced increased expression of antiviral genes and reduces their basal expression. RAW 264.7 cells were incubated in the absence (DMSO) or presence of the JAK inhibitor ruxolitinib for 6 h at 37°C and then at the indicated temperature for 12 h. Expression of the indicated genes was analyzed by RT-qPCR. mRNA expression is relative to Hprt (*n* = 3, mean ± SD). Statistical significance was determined by unpaired *t*-tests and is indicated by asterisks: *P< 0.05; **P< 0.01; ***P< 0.001; ns = not significant.

Almost all factors involved in the canonical antiviral pathway displayed increased expression at 38°C in our RNA-seq data (Figure 2A, only genes depicted in gray did not show a significant change). To confirm this finding, RAW 264.7 cells were incubated at 35, 37, 38 and 39°C, and the expression of genes implicated in the defense against RNA viruses, including but not limited to SARS-CoV-2 (17,22), was analyzed. RT-qPCR confirmed temperature-controlled expression of RIG-I (*Ddx58*), the sensor of viral RNA, IRF7, one of the transcription factors that induces type I interferons, STAT2 and a variety of interferon-stimulated genes (ISGs); the validation rate was 100%, demonstrating a robust sequencing and analysis pipeline as well as biological reproducibility. Notably, all RT-qPCRs show the same pattern, a strong upregulation of the respective gene when increasing the temperature from 37 to 38°C (Figure 2A). A further temperature increase to 39°C did not substantially increase the expression of these temperature-controlled genes, indicating that they are controlled through slightly elevated temperature and that this is different from the response during a high fever. Similarly, the decrease to 35°C had only a small effect, indicating that this regulation takes place in the normothermic range. To further test this, we used RAW 264.7 cells that were incubated at 36.5, 37, 37.5 and 38°C for 12 h. Remarkably, for three out of four tested targets we observe a significant increase in expression already when increasing the temperature from 36.5 to 37.5°C with the fourth target gene showing a similar trend (Figure 2B). These data show that body temperature changes in the sub-pathological range, for example dependent on individual variation, circadian time or age, can control the expression of antiviral genes, with higher temperature leading to higher expression. These results together suggest that antiviral immunity is increased within a very narrow temperature window in the normothermic range, when body temperature rises above 37°C, and that it reaches its maximum increase at 38°C. Although our data clearly show that an increase from 36.5 to 37.5°C activates this pathway, we have compared 37 and 39°C in the following experiments, to accommodate a 0.5°C degree error margin of the incubators used for the experiments.

Given the prominent role of JAK-STAT signaling in this process, we addressed a potential increase in JAK-STAT signaling at higher temperature. We used phospho-STAT2/3 antibodies to investigate STAT activation at normothermia and increased temperature. Indeed, an increase in temperature alone, without any additional stimulation, was sufficient to increase phosphorylation, and thus activation, of STAT2 and STAT3 in RAW 264.7 cells (Figure 2C and Supplementary Figure S2A). Notably, STAT2 phosphorylation at higher temperature was comparable with the level obtained by LPS stimulation at 37°C, showing the strong impact of slightly increased temperature. As activation of the JAK-STAT pathway is critical for the activation of ISGs, these data again suggest that a subtle increase in temperature primes macrophages for an antiviral response. It is noteworthy that we also observe an increase in STAT2 and RIG-I protein, confirming that the temperature-controlled changes observed for mRNAs (Figures 1 and 2A) are translated to the protein level (Figure 2D and E). For STAT2, we observed increased levels at higher temperature mainly in the nuclear fraction, indicating activated STAT2, with a similar tendency in the cytoplasm (Supplementary Figure S2B). To further address the role of JAK-STAT signaling in temperature-controlled expression of antiviral genes, we applied the JAK inhibitor ruxolitinib at different temperatures. Ruxolitinib not only completely prevented the temperature-controlled increase in antiviral genes but also reduced their basal expression at 37°C (Figure 2F). In addition to reduced expression of direct ISGs such as *Oasl2* and *Ifit3*, we observed reduced expression of *Trim25*, *Ddx58* (RIG-I), *Irf7* and *Stat2* upon treatment with ruxolitinib, suggesting that also upstream mediators of the pathway are under control of JAK-STAT signaling. These data are consistent with a model in which increased JAK-STAT signaling at elevated temperature forms the basis for increased expression of antiviral genes. This could be mediated through basal JAK-STAT activity and a temperature-mediated increase in STAT2 expression, which would activate a feed-forward mechanism that increases the expression of antiviral genes and STAT2 itself.

### Elevated temperature increases antiviral genes and NO production in primary mouse macrophages

**Figure 3.**
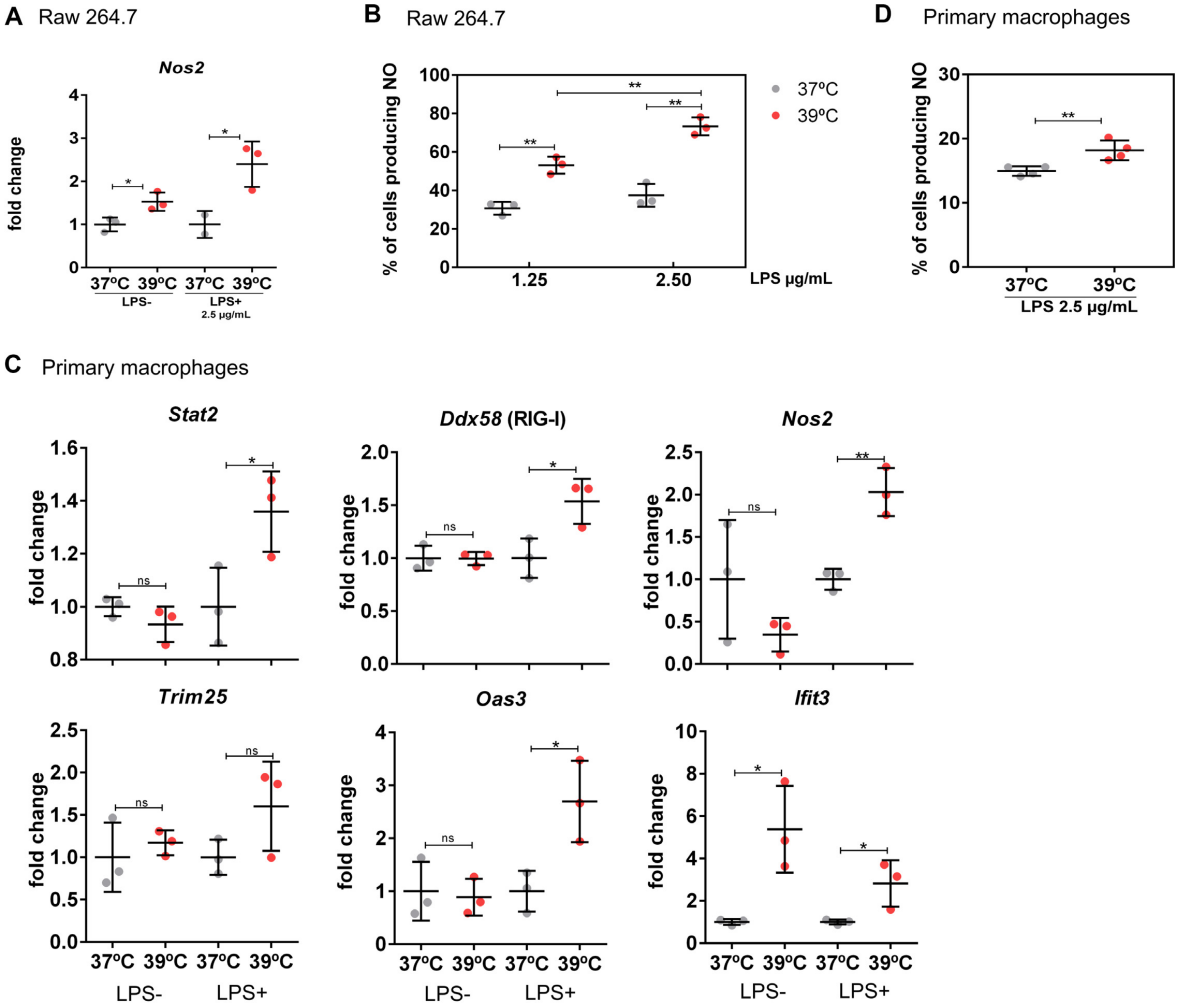
Increased temperature induces NO production and antiviral genes also in primary macrophages. **(A)** A second antiviral mechanism, production of NO, is increased at elevated temperature in RAW 264.7 cells. mRNAs are normalized to 37°C of the respective treatment. Cells were incubated at the indicated temperature for 12 h in the absence or presence of LPS and the expression of *Nos2* was analyzed by RT-qPCR. Mean values ± SD are shown (*n* = 3). **(B)** RAW 264.7 cells were treated as indicated and NO-producing cells were detected by flow cytometry (see the ‘Materials and Methods’ section). Shown is the percentage of NO-producing cells (mean ± SD, *n* = 3). **(C)** Increased temperature induces antiviral genes in activated primary macrophages. Primary mouse bone marrow-derived macrophages were incubated at different temperatures for 16 h in the absence or presence of LPS (0.1 μg/ml) as indicated. Expression of antiviral genes was analyzed by RT-qPCR. mRNAs are normalized to 37°C of the respective treatment (mean ± SD, *n* = 3). **(D)** NO production was analyzed in primary macrophages as in panel (B) (mean ± SD, *n* = 3). Statistical significance was determined by unpaired *t*-tests and is indicated by asterisks: *P < 0.05; **P < 0.01; ns = not significant.

In addition to the activation of type I interferon signaling, production of NO is another defense mechanism against viral infection (23,24), also in the context of SARS-CoV-2 (24). Our RNA-seq data showed an around 2-fold increase of *Nos2*, the inducible enzyme producing NO in macrophages, when increasing the temperature from 37 to 38°C. We confirmed this regulation in RAW 264.7 cells using independent RT-qPCRs. The effect of temperature was even stronger, when cells were pre-incubated at the different temperatures and then stimulated with LPS (Figure 3A). This suggests that macrophages at slightly higher temperature are in a primed condition that, with respect to NO production, allows stronger activation. To support this hypothesis, we used the same experimental setup, pre-incubation at 37 or 39°C followed by LPS treatment at the respective temperature, and measured NO production directly. We observed a substantial and significant increase in the number of NO-producing cells (Figure 3B) and the amount of produced NO (Supplementary Figure S3A) confirming higher activity at higher temperature also of this branch of the antiviral defense.

We then aimed to confirm the impact of temperature on antiviral response mechanisms in primary cells. To this end, we prepared primary mouse macrophages from bone marrow, incubated them at different temperatures for 16 h and then performed RT-qPCR. Except for *Ifit3*, primary mouse macrophages did not show differential expression of antiviral response genes when treated with different temperatures alone, but showed a substantial increase of key antiviral response genes at higher temperature, when additionally treated with a low dose of LPS (0.1 μg/ml) (Figure 3C). This is consistent with a previous report suggesting that RAW 264.7 cells are in a chronically activated state (25) and points to a model in which temperature and an additional stimulus act together for full activation of the pathway (Supplementary Figure S3). Interestingly, we find a similar situation in human Jurkat T cells, which show temperature-controlled expression of antiviral genes not in the basal but only in the activated state (Supplementary Figure S3C). This suggests a general mechanism conserved across cell types and species, which uses elevated temperature to prime immune cells for stronger activation. We also measured NO production when primary macrophages were LPS stimulated at different temperatures and observed a small but significant increase in NO-producing cells and NO production at the higher temperature (Figure 3D and Supplementary Figure S3B). Together, these data show that in macrophages two critical antiviral pathways, type I interferon response and NO production, are primed by elevated temperature and that these mechanisms are also active in primary macrophages.

### Temperature-controlled STAT2 pre-mRNA processing can induce transcription of antiviral genes at elevated temperature

**Figure 4.**
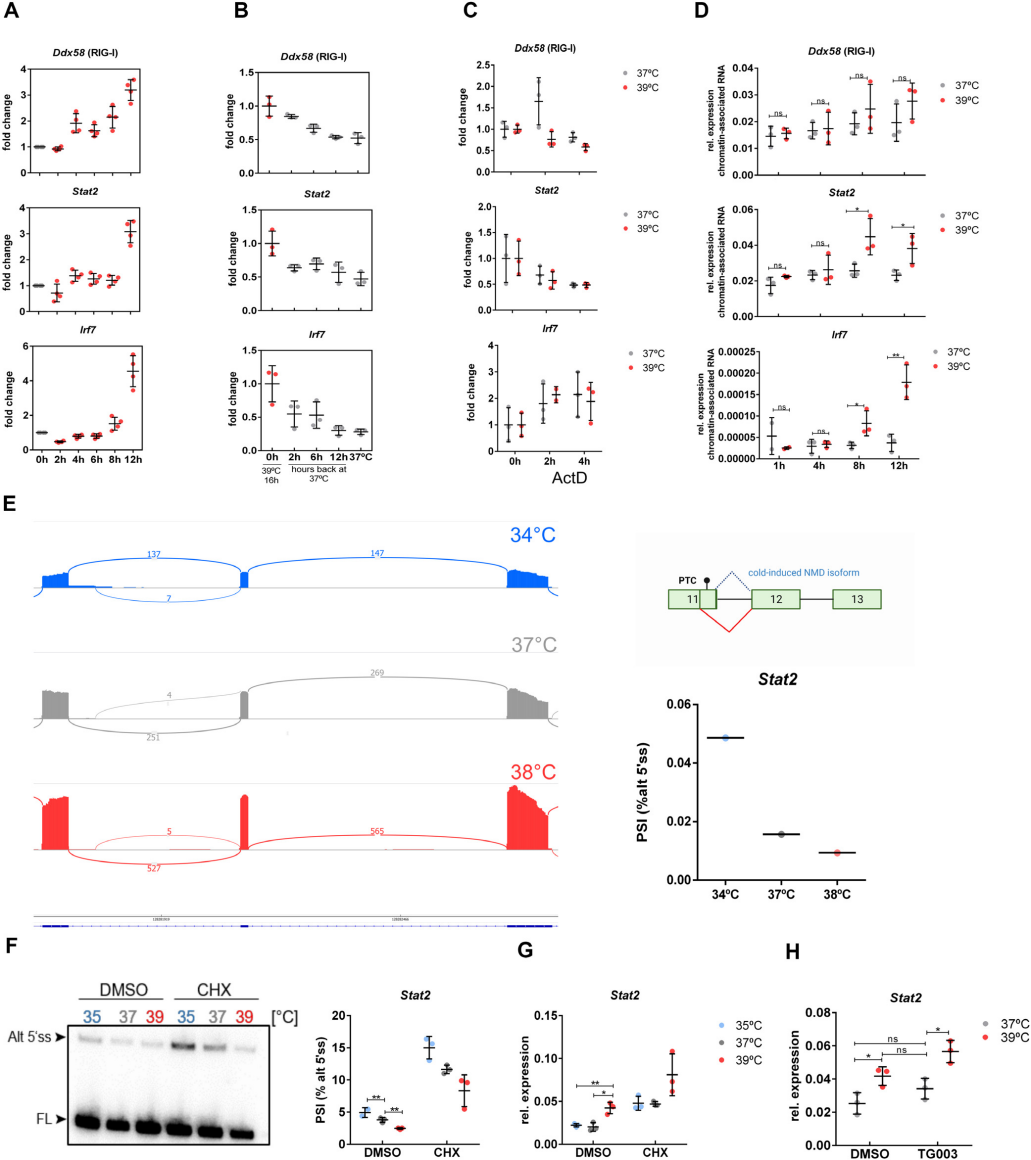
*Stat2* alternative splicing coupled to nonsense-mediated decay (NMD) provides a molecular link between elevated temperature and increased *de novo* transcription of antiviral genes. **(A)** Delayed increase of antiviral gene expression upon temperature increase. RAW 264.7 cells were incubated at 39°C for the indicated time and RNA was analyzed by RT-qPCR. mRNAs are normalized to 37°C (mean ± SD, *n* = 3). **(B)** The temperature-induced increase in antiviral gene expression is reversible. RAW 264.7 cells were incubated for 16 h at 39°C and then transferred to 37°C for the indicated time. RNA was prepared and analyzed by RT-qPCR (mean ± SD, *n* = 3). mRNAs are normalized to 39°C. **(C)** RNA stability of antiviral genes is not altered at increased temperature. RAW 264.7 cells were pre-incubated at the indicated temperature for 12 h. ActD was then added and mRNA was quantified 2 and 4 h later. mRNAs are normalized to *t* = 0 of the respective temperature (mean ± SD, *n* = 4). **(D)** Increased *de novo* transcription of antiviral genes at increased temperature. RAW 264.7 cells were incubated at 39°C for the indicated time; chromatin-associated RNA was purified and analyzed by RT-qPCR with the forward primer binding to an intronic region (for *Irf7* the forward primer binds in an exonic region). mRNA expression is relative to Hprt. **(E)** Sashimi plot (left) identifying an alternative, NMD-inducing 5′ splice site in *Stat2* exon 11, which is used more frequently at 34°C and shows reduced usage at 38°C. Reads from the sashimi plot are quantified (right, bottom). Schematic representation of the NMD-inducing 5′ splice site in *Stat2* exon 11 (right, top). Created with BioRender.com. **(F)** Radioactive, splicing-sensitive RT-PCR confirming decreased use of the alternative 5′ splice site at warmer temperature and stabilization of the alternative isoform in the presence of the NMD inhibitor CHX. RAW 264.7 cells were incubated at the indicated temperature for 8 h, and then for an additional 4 h in the absence (DMSO) or presence of CHX. RNA was prepared and analyzed by RT-PCR. A representative gel (left) and phosphorimager quantification (right, mean ± SD, *n* = 3) are shown. **(G)** Samples as in panel (F) were analyzed by RT-qPCR. mRNA expression is relative to Hprt. **(H)** The CLK inhibitor TG003 has a similar effect to the CHX treatment (mean ± SD, *n* = 3). RAW 264.7 cells were incubated for 6 h in the absence (DMSO) or presence of the CLK inhibitor TG003, and then for an additional 12 h at the indicated temperature. mRNA expression is relative to Hprt (mean ± SD, *n* = 3). Statistical significance was determined by unpaired *t*-tests and is indicated by asterisks: *P < 0.05; **P < 0.01; ns = not significant.

To address the mechanistic basis for increased expression of antiviral genes at higher temperature, we first investigated the kinetics of changes in gene expression. We observed a delayed response to a shift in temperature, as a substantial and significant increase in expression was only observed after 12 h at higher temperature (Figure 4A). This temporal profile suggests that the increased expression is not directly coupled to changes in temperature, as alternative splicing events that are controlled through CLK-mediated SR protein phosphorylation start to change much faster (4), but rather suggest that an intermediate step is required. We then tested whether and how fast the increased expression at higher temperature was reversible. When cells were switched back from 39 to 37°C, expression of antiviral genes steadily declined and reached their basal level after around 12 h (Figure 4B). The kinetics was slightly different for different targets, e.g. the decline of *Stat2* mRNA appeared to be faster, which likely reflects individual differences in mRNA turnover. Together, temperature-controlled changes in antiviral genes show a delayed increase at elevated temperature, which is reversible upon restoration of lower temperature.

We then considered changes in mRNA stability or *de novo* transcription as processes that could form the basis for the temperature-controlled change in antiviral gene expression. To determine mRNA stability of antiviral genes, we treated RAW 264.7 cells at different temperatures with ActD and quantified the remaining mRNA at several time points after addition of ActD. We did not observe strongly altered stability of mRNAs when comparing cells at 37 and 39°C, suggesting that regulated mRNA stability is not the cause for temperature-controlled expression (Figure 4C). As an approximation for *de novo* transcription, we quantified chromatin-associated, intron-containing RNA. These analyses showed a clear trend toward increased expression after 8 and 12 h at warmer temperature (Figure 4D), resembling the kinetics of accumulation of the respective mRNAs (Figure 4A). We thus conclude that increased expression of antiviral genes at increased temperature is mediated, at least in part, through increased *de novo* transcription.

As our inhibitor and phosphorylation experiments (Figure 2C and F) indicated that increased expression of *Stat2* would be sufficient to activate the antiviral response at higher temperature, we further investigated the mechanistic basis of increased *Stat2* expression. Examining *Stat2* RNA-seq reads at 34, 37 and 38°C revealed the presence of a so far not annotated temperature-dependent alternative 5′ splice site in exon 11. Usage of this alternative 5′ splice site was higher at lower temperature and leads to a frameshift and the inclusion of a premature stop codon (PTC), thus preventing the formation of a functional protein and leading to NMD (Figure 4E). In line with this conclusion, we find that blocking NMD by the addition of CHX increases the abundance of the PTC-containing isoform and total *Stat2* mRNA (Figure 4F and G). Addition of CHX at 37°C increases *Stat2* mRNA to the level observed at 39°C in control cells, pointing to alternative splicing coupled to NMD as the main mechanism regulating temperature-dependent *Stat2* expression (Figure 4G). Temperature-controlled *Stat2* alternative splicing coupled to NMD is further supported by independent RNA-seq datasets from mouse hepatocytes (26), demonstrating that the alternative *Stat2* isoform and total *Stat2* mRNA strongly increase by inhibiting NMD through addition of CHX (Supplementary Figure S4A and B) and RNA-seq data from MEFs showing increased *Stat2* expression at higher temperature (27) (Supplementary Figure S4C). In addition, we find several NMD-inducing *STAT2* splicing isoforms and temperature-controlled *STAT2* expression in a human cell line (Supplementary Figure S4D and E), confirming that the mechanism, temperature-controlled alternative splicing coupled to NMD leading to increased *STAT2* expression at higher temperature, is conserved across species.

We have previously shown that CLKs act as direct temperature sensors to translate changes in the physiologically relevant temperature range into changes in alternative splicing and gene expression (9,28). Interestingly, antiviral genes, including *Stat2*, at 37°C show a trend toward increased expression in the presence of the CLK inhibitor TG003 (Figure 4H and Supplementary Figure S5). This is consistent with our previous finding that CLKs are less active at higher temperature and the notion that inhibition of CLKs at 37°C mimics the effect of warmer temperature (9). However, as the expression of antiviral genes in the presence of TG003 is further increased at 39°C (Figure 4H and Supplementary Figure S5), additional mechanisms are likely involved in this regulation. Altogether, the data are consistent with a model in which CLKs contribute to temperature-controlled *Stat2* expression, which, at elevated temperature, increases expression of ISGs and further antiviral genes to increase the defense against viral infection.

**Figure 5.**
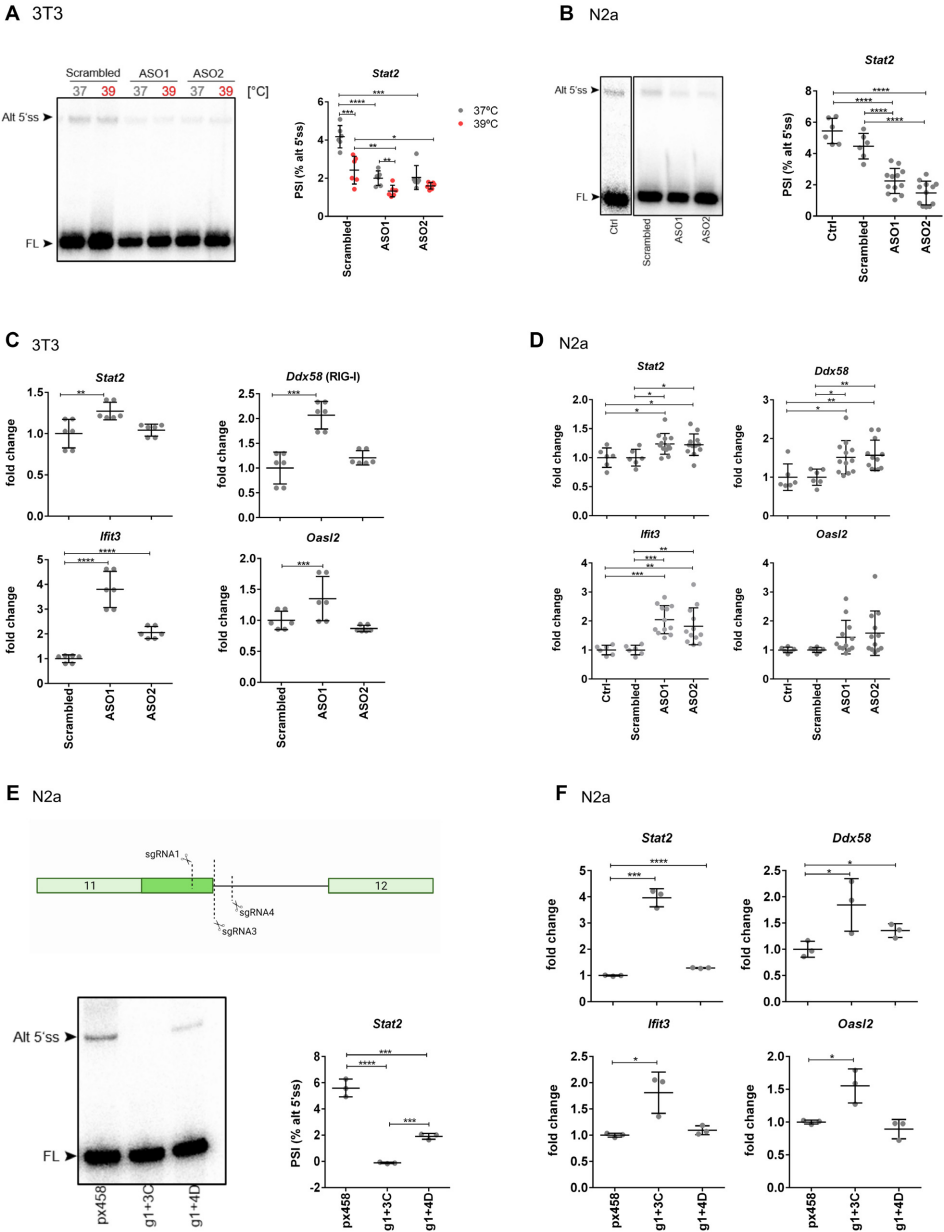
Deletion or reduced usage of the alternative 5′ splice site of exon 11 in Stat2 increases the expression of antiviral genes. **(A)** 3T3 cells were transfected with two different ASOs targeting the alternative 5′ splice site in Stat2 exon 11. Thirty-two hours post-transfection, cells were incubated for 16 h at 37 or 39°C. A scrambled ASO was used as a control. Radioactive, splicing-sensitive RT-PCR confirms decreased use of the alternative 5′ splice site at warmer temperature and in the presence of the ASOs. A representative gel (left) and phosphorimager quantification of two independent experiments performed in triplicates (right, mean ± SD, *n* = 6) are shown. **(B)** Radioactive, splicing-sensitive RT-PCR confirms decreased use of the alternative 5′ splice site in the presence of the ASOs in N2a cells. A representative gel (left) and phosphorimager quantification of several independent experiments (right, mean ± SD, Ctrl and scrambled = 6, ASOs = 12) are shown. Empty transfection (Ctrl) and a scrambled ASO were used as a control. **(C)** Gene expression levels of the antiviral genes upon ASO treatment in 3T3 cells. Gene expression was investigated by RT-qPCR. mRNAs are normalized to scrambled ASO at 37°C (mean ± SD, *n* = 6). **(D)** Same as in panel (C) for N2a cells (mean ± SD, Ctrl and scrambled = 6, ASOs = 12). **(E)** Depletion of the alternative 5′ splice site of exon 11 using CRISPR/Cas9 in N2a cells (top: position of the sgRNAs; created with BioRender.com); bottom: a radioactive RT-PCR investigating Stat2 AS in a homozygous clone (g1 + 3C) and a heterozygous clone (g1 + 4D) is shown and quantified (right, mean ± SD, *n* = 3). **(F)** Increased expression of Stat2 and other antiviral genes in cells lacking the alternative 5′ splice site. Gene expression was investigated by RT-qPCR. mRNAs are normalized to px458 as nonedited control (mean ± SD, *n* = 3). Statistical significance was determined by unpaired *t*-tests and is indicated by asterisks: *P< 0.05; **P< 0.01; ***P< 0.001; ^****^P< 0.0001.

To further validate the influence of the alternative 5′ splice site of exon 11 in *Stat2* on the expression of antiviral genes, we used ASOs that allow direct manipulation of *Stat2* AS. We used 3T3 and N2a cells, which were transfectable with the ASOs. Transfection of two different ASOs targeting the alternative NMD-inducing 5′ splice site led to a significant reduction of the usage of this splice site (Figure 5A and B). Further, gene expression analyses showed an increased expression of *Stat2* and other ISGs in the ASO-treated cells (Figure 5C and D). In addition, the effect of the ASOs on the expression of ISGs resembles the effect of higher temperature in 3T3 cells (Supplementary Figure S6). Similarly to RAW 264.7 cells, 3T3 cells showed increased expression of antiviral genes at 39°C when compared to 37°C (Supplementary Figure S3E) and a corresponding decreased usage of the alternative 5′ splice site at 39°C (Figure 5A).

To independently validate the observed effect, we generated a CRISPR/Cas9-edited N2a cell line lacking the alternative NMD-inducing 5′ splice site. Either homozygous or heterozygous removal of the respective genomic sequence was confirmed by radioactive RT-PCR at RNA level (Figure 5E). As expected, we observed higher *Stat2* expression in the homozygous cell line, when compared with a nonedited control and a small but significant increase in the heterozygous cell line (Figure 5F). We then addressed the impact of the depletion of the alternative 5′ splice site on the expression of other antiviral genes. The expression of *Ddx58* was significantly increased in both cell lines and expression of *Oasl2* and *Ifit3* was significantly increased in the homozygous cell line, compared to the nonedited control (Figure 5F). Taken together, these data show that alternative splicing coupled to NMD regulates temperature-dependent *Stat2* expression, and can induce the transcription of antiviral genes.

### Genes that restrict SARS-CoV-2 infection show higher expression at higher temperature and reduced expression with aging

**Figure 6.**
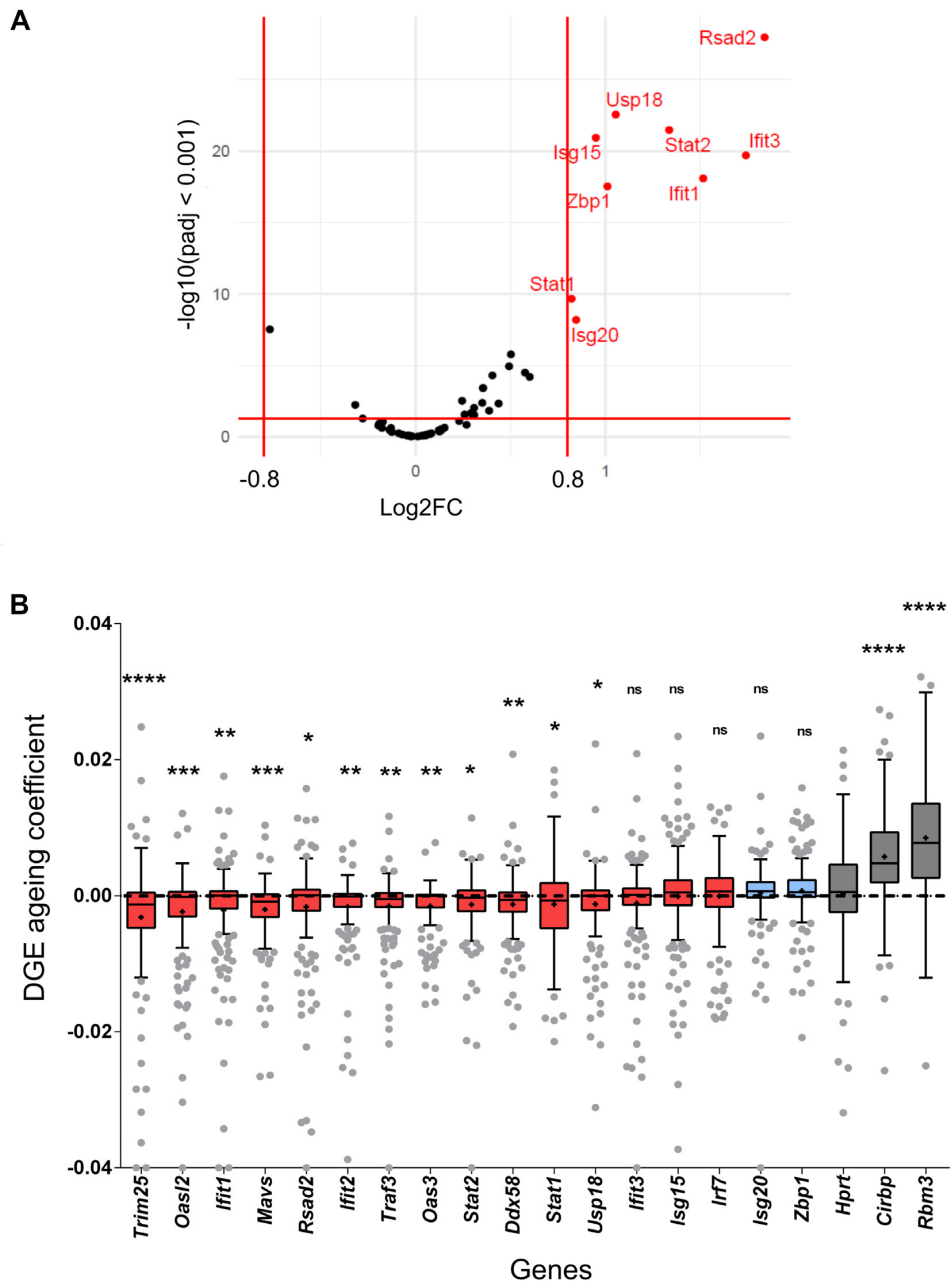
The expression of SARS-CoV-2 restriction factors is controlled by temperature and increased in children compared to seniors. **(A)** The expression of 65 SARS-CoV-2 restriction factors from (29) was analyzed in our RNA-seq data from RAW 264.7 cells and compared between 37 and 38°C. In the volcano plot, log_2_ fold change was plotted against *P*_adj_ < 0.001 (Benjamini–Hochberg adjusted *P*-values); fold changes were considered significant if log_2_ fold change >0.8. Genes upregulated at 38ºC are shown in red. **(B)** Warm-induced antiviral genes and SARS-CoV-2 restriction factors show decreased expression with increasing age. DGE data for the indicated genes in 24 tissues (193 cell types) from 1- and 30-month-old mice are compared. Indicated is the ‘aging coefficient’ as defined in (30). The aging coefficient is shown for all analyzed tissues. The box plot shows the interquartile range (IQR) divided by the median, and Tukey-style whiskers extend to a maximum of 1.5 × IQR beyond the box; mean values are indicated by + symbols. Values were sorted by the mean (red = negative mean values, corresponding to reduced expression in old individuals; blue = positive mean values). Each gene was compared to *Hprt* DGE and statistical significance was determined by unpaired *t*-tests and is indicated by asterisks: *P< 0.05; **P< 0.01; ***P< 0.001; ^****^P< 0.0001; ns = not significant. The cold-induced RNA binding proteins *Rbm3* and *Cirbp* serve as control; their increased expression in old individuals indicates reduced body temperature with aging.

While the mechanism we describe here is relevant for infection with diverse viruses, we focused on SARS-CoV-2 infection to address the functionality of temperature-controlled antiviral immunity *in vivo*. We first used published data that identified 65 cellular factors that restrict SARS-CoV-2 infection (29). Notably, nine of these SARS-CoV-2 restriction factors showed a highly significant and substantial (log_2_ fold change >0.8) increase in expression in our RNA-seq data when comparing 37°C with 38°C and many more show a more subtle increase in expression at higher temperature (Figure 6A). In contrast, no SARS-CoV-2 restriction factor showed higher expression at lower temperature when applying the same thresholds (Figure 6A). These data strongly suggest that increasing temperature from 37 to 38°C creates a condition that limits SARS-CoV-2 infection. This could be especially relevant during aging, as children have a higher body temperature than adults, which then further declines in the elderly. This correlates with much higher susceptibility for severe SARS-CoV-2 infections in seniors (with reduced body temperature) than is observed for children (with higher body temperature). To correlate the expression of antiviral genes with aging, we used a single-cell RNA-seq dataset from different mouse tissues sequenced at 1 and 30 months of age (30), corresponding to early childhood and 100 years of age in humans. In line with our hypothesis, antiviral genes that are warm induced in our data showed increased expression in younger animals (Figure 6B). This is a global effect, as we used a mean value of all analyzed tissues and all cell types within the different tissues. The regulation is therefore likely caused by a systemic signal such as body temperature and not by cell- or tissue-specific signaling events. While the fold change of the individual genes is small, we hypothesize that the combined effect of many small changes in the same pathway will have a considerable combined effect on antiviral immunity. Importantly, *Rbm3*, which is cold induced and reacts extremely robust and sensitive to changes in temperature (Supplementary Figure S1A), shows higher expression in older animals, and so does another cold-induced RNA binding protein, *Cirbp* (9) (Figure 6B), which argues for reduced body temperature in the older animals. These data are line with the idea that higher body temperature of young animals increases the expression of antiviral response genes. This, however, remains a hypothesis based on a correlation that awaits direct experimental proof, e.g. by acutely manipulating the body temperature of aged mice and investigating the impact on antiviral immunity. We also note that aging is a multifactorial process and altered body temperature very likely is only one of several factors that affects the expression of antiviral genes in aged individuals. Importantly, a recent study in humans also suggested higher antiviral innate immunity in children compared to adults (19). Our data now provide a possible mechanistic link between higher body temperature in children versus seniors and higher expression of antiviral genes in children.

### SARS-CoV-2 infection is less efficient at higher temperature in cell culture and *in vivo*

**Figure 7.**
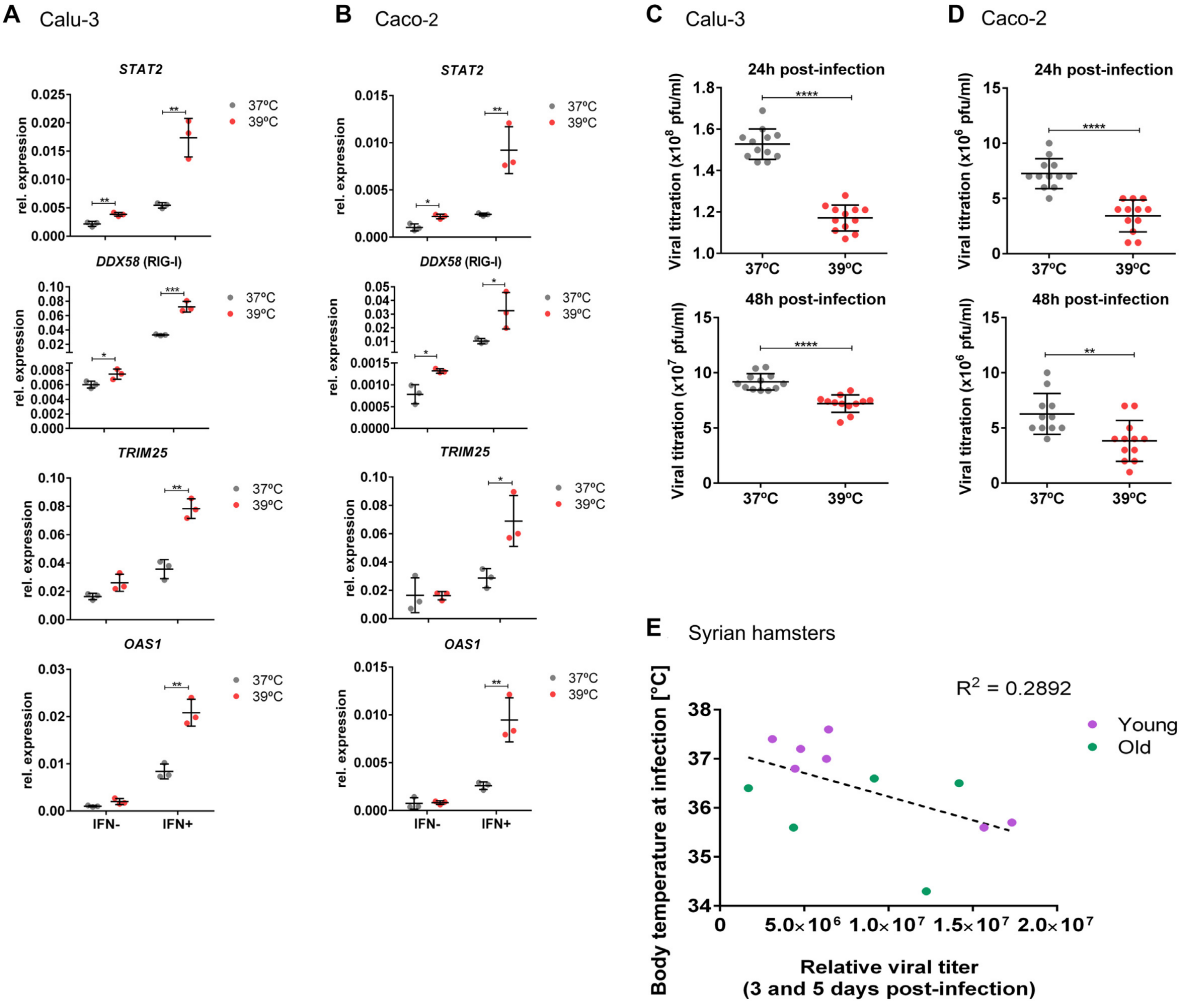
SARS-CoV-2 replication is reduced by pre-incubation at warmer temperature in cell culture and *in vivo*. **(A)** Calu-3 cells show increased expression of antiviral genes at 39°C. Cells were incubated at the indicated temperature for 12 h, and then for 4 h ± IFN (100 U/ml). RNA was extracted and analyzed by RT-qPCR. mRNA expression is relative to GAPDH. Mean values ± SD are shown (*n* = 3). **(B)** Same as in panel (A) for Caco-2 cells. **(C)** Pre-incubation at 39°C for 16 h reduced viral replication. Calu-3 cells were pre-incubated at 37 or 39°C for 16 h and then infected with SARS-CoV-2, following incubation at 37°C for 24 and 48 h. The viral titer as determined by the plaque assay is reduced in cells that were pre-incubated at 39°C. Mean values ± SD are shown (*n* = 12). (**D)** Same as in panel (C) for Caco-2 cells. (**E)** Published data from (31) were used to plot the body temperature of Syrian hamsters at the time point of SARS-CoV-2 infection against the viral mRNA detected in lungs 3 or 5 days post-infection (see Supplementary Figure S7 for matched subgroups). Statistical significance was determined by unpaired *t*-tests and is indicated by asterisks: *P< 0.05; **P< 0.01; ***P< 0.001; ^****^P< 0.0001.

To confirm an impact of temperature on SARS-CoV-2 infection, Calu-3 and Caco-2 cells were used. Cells were pre-incubated at 37 or 39°C for 16 h, as this was the time frame required to induce antiviral genes (Figure 4A), and then infected with SARS-CoV-2 and incubation was continued at 37°C for 24 and 48 h. Calu-3 and Caco-2 cells showed increased expression of *STAT2* and *DDX58* after 16 h pre-incubation at 39°C when compared with the 37°C control (Figure 7A and B). Further stimulation with type I IFN increased the expression of other antiviral genes, such as *OAS1* and *TRIM25*, at 39°C. These data support the idea that an increase in *STAT2* expression can induce the cascade and lead to a primed state, which can then be triggered by an additional stimulus for full activation. We then determined the viral titer 24 and 48 h post-infection. Higher viral loads were observed in cells that were kept at 37°C when compared with the cells that were pre-incubated at 39°C (Figure 7C and D). This experiment confirms that a slightly increased temperature during a 16 h time window prior to infection can slightly but significantly reduce viral replication.

Finally, to address a connection of body temperature with SARS-CoV-2 infection *in vivo*, we used a cohort of hamsters that were infected with SARS-CoV-2, in which the body temperature at the time point of infection was measured (31). Viral titers were then assessed 3 or 5 days post-infection and plotted against the body temperature at the time point of infection (Figure 7E). This analysis suggests that animals with higher body temperature at the time of infection can control the viral infection better than animals with lower body temperature, as we observe a correlation of higher body temperature with reduced viral titer several days post-infection (Figure 7E and Supplementary Figure S7). This study has clear limitations, as we analyzed a diverse cohort of hamsters (different age, different time point after infection); in addition, we have not determined for how long prior to infection the animals displayed the respective temperature. We note that focusing on a smaller subgroup of hamsters, e.g. only young animals or only one time point after infection, increases the correlation of higher body temperature at the time point of infection with reduced viral load (Supplementary Figure S7A–C), but larger *in vivo* studies are required to increase statistical power. Taken together, our data provide evidence for an antiviral mechanism that is set in motion through temperature-controlled NMD-inducing *STAT2* alternative splicing, which protects against viral infection at higher body temperature and may contribute to differential susceptibility of children versus seniors toward SARS-CoV-2 infection.

## DISCUSSION

**Figure 8.**
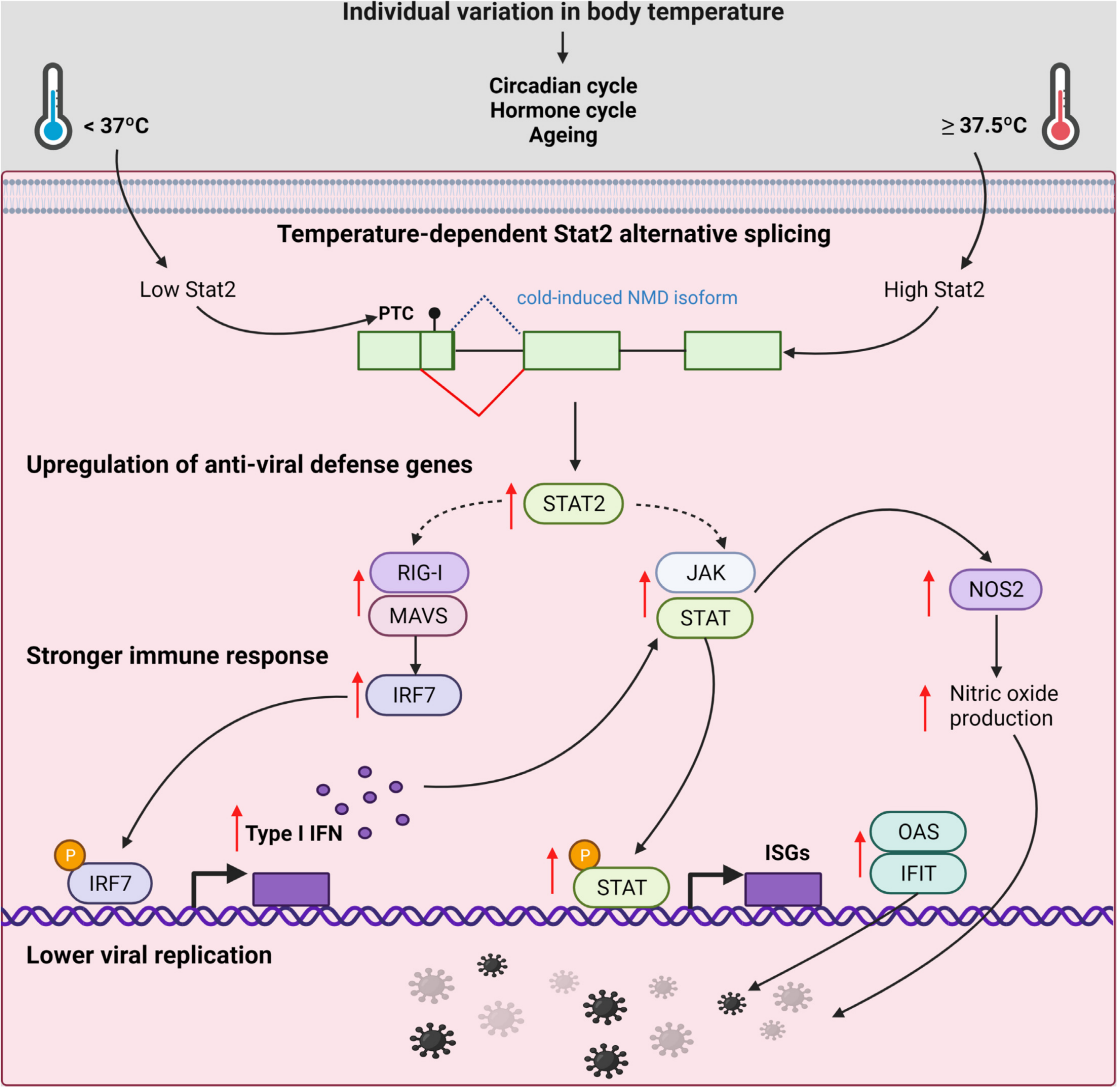
Schematic representation of the impact of Stat2 on the expression of antiviral genes at higher temperatures. Alternative splicing coupled to NMD decreases Stat2 expression in colder conditions. An increase of Stat2 expression at warmer temperatures together with JAK activity will induce the expression of the antiviral genes. This will then lead to a stronger antiviral immune response and as a consequence a lower viral replication. Created with BioRender.com.

There is ample evidence showing that an increase in core body temperature of 1–4°C during fever is linked to improved survival and the resolution of many infections (10). However, little is known about the temperature-sensing machinery that triggers changes in immune cell behavior. In addition, individual variation or circadian and age-dependent changes in core body temperature in the sub-pathological range have not been considered when addressing innate immunity. Here, we show that subtle changes in body temperature control the expression of innate immune genes in a JAK-STAT-dependent manner, which contributes to altered vulnerability to viral infections, including SARS-CoV-2, already in the sub-pathological temperature range. Our data provide evidence for body temperature-controlled regulation of antiviral immunity with broad implications for the understanding of susceptibility to viral infection, the biology of aging and other conditions with altered core body temperature, such as the female hormone cycle or prolonged fasting. The change in core body temperature in these settings is subtle, typically within a 1°C window. We show that even these small temperature changes can act as systemic signal to increased expression of antiviral genes, as the upstream signaling pathway is remarkably sensitive and reacts exactly in the sub-pathological temperature range provided by these conditions (Figure 8).

The mechanism we describe requires a prolonged period at elevated temperature, as we observe the upregulation of antiviral genes only 12 h after temperature change. Short-term increase in core body temperature, as observed e.g. during physical exercise, therefore seems not to be sufficient to increase antiviral immunity. This delay likely acts as safety mechanism to avoid unnecessary and potentially harmful activation of the interferon response and NO production. The circadian body temperature profile with roughly 12 h at a higher temperature followed by 12 h of decreased temperature falls within the time frame that could control expression of antiviral genes. This will, however, depend on individual body temperature rhythms that in many cases will deviate from the textbook 12 h periods. It is worth noting that mice control a viral infection better when infected during the active phase, which could be related to higher body temperature (32). Similarly, female mice have been suggested to better control viral infections in the luteal phase of the hormone cycle, which is also the phase with higher body temperature (5).

The reason for the delayed response is that the expression of antiviral genes is not directly coupled to temperature changes but linked through an intermediate step of pre-mRNA processing. Our data are consistent with a model in which CLKs act as temperature sensors to control *Stat2* pre-mRNA processing as an upstream event. However, the extremely narrow temperature window between 36.5 and 38°C in which the expression of antiviral genes is controlled is very different from the temperature-dependent change in CLK activity we observe *in vitro* (9). It is therefore likely that additional mechanisms contribute to the regulation of antiviral genes, to achieve the extraordinary temperature sensitivity. We show that temperature-controlled expression of antiviral genes is dependent on JAK-STAT signaling, as it is completely abolished in the presence of ruxolitinib. In addition, we show that *Stat2* expression is increased at higher temperature through reduced alternative splicing coupled to NMD, and can then activate the type I interferon response. This model is consistent with previous work suggesting that manipulating STAT2 levels is sufficient to control antiviral immunity (33). This model may also explain why we observe that in some cell types, such as primary macrophages, temperature alone is not sufficient for activation. An increase of *Stat2* alone will not activate the pathway but requires additional JAK activity. In the absence of basal JAK activity, an increase in temperature will increase *Stat2* levels, which would represent a primed state. A further activating stimulus that induces JAK activity can then induce a stronger activation of the type I interferon response, which is what we have observed for primary murine macrophages and human Jurkat T cells. This would also be highly beneficial *in vivo* during viral infections. While the body temperature at the time of infection (and 12 h before) will influence the efficiency of the immediate reaction, the increase in body temperature induced by the initial encounter with the virus will prime cells of the innate immune system, including but not limited to cells that migrate toward the area of infection, for a more efficient activation of the type I interferon pathway.

Earlier work has shown that human rhinovirus, the common cold virus, replicates better at 32°C, in the upper respiratory tract than in the lung at 37°C core body temperature. This may be due to a more efficient innate immune response at 37°C (34). Similarly, human macrophages have been suggested to be more active at febrile temperatures (10). In mice, febrile temperatures lead to an increase in LPS-induced downstream signaling (25), which is in line with our model that higher temperature primes the innate immune system for stronger activation. In mouse macrophages, febrile temperature also increases NO production in a JAK-STAT-dependent manner (35). NO has a broadly antiviral activity and NO-based therapies have also been discussed in the context of SARS-CoV-2 infections (24). Given that we find both the type I interferon response and NO production to be dynamically controlled in the narrow temperature window of healthy human core body temperature, we suggest that even subtle temperature fluctuations can have a substantial impact on the outcome of viral infections. This conclusion is supported by our *in vivo* data suggesting a correlation of higher hamster core body temperature at the time of infection with reduced SARS-CoV-2 replication. Our cell culture experiments further support this model by showing that a 16 h pre-incubation at higher temperature reduced SARS-CoV-2 replication. While this appears to be a subtle effect, it may still contribute to determine the course of an infection *in vivo*. These data together show that innate immunity is controlled through alterations in the window of healthy body temperature. Our work thus closes a gap in understanding the impact of temperature on innate immunity, as previous work has focused on temperatures below 35°C (upper respiratory tract) or at fever above 38°C, while the healthy body temperature range was so far not taken into consideration. Concerning the upper respiratory tract, it has long been suggested that prewarming breathing air in winter, e.g. by breathing through a scarf or a face mask, can reduce viral infections (36), which could be related to the mechanism we describe here.

It is well established that body temperature decreases with aging. On average, healthy elderly people have a lower body temperature when compared with younger adults and children (2,6). The level of physical activity is also lower in seniors than in children, which further contributes to a reduction of average core body temperature. Additionally, elderly people usually fail to reach the fever temperature range, and therefore are less capable of mounting a strong inflammatory response to infection and disease (2). A possible consequence of reduced body temperature in older individuals could be altered CLK activity and SR protein phosphorylation, which, however, needs to be experimentally confirmed. Consistent with our model, recent work showed an enhanced innate antiviral capacity in children, leading to an efficient early production of interferons in the SARS-CoV-2-infected upper airways (19). This situation likely contributes to the better protection of children against a severe course of COVID-19 (37,38) and their overall lower susceptibility to SARS-CoV-2 infection (39). Furthermore, the expression of many of the genes that we find controlled by temperature changes, for example *DDX58* (RIG-I), *IFIT1-3*, *OAS1-3* and *OASL*, was found to be reduced in >60 years old versus <60 years old SARS-CoV-2-positive subjects (40). These findings are in line with our model that age-dependent body temperature variation controls the expression of innate immune genes and SARS-CoV-2 replication. Altogether, our data may contribute to a mechanistic explanation why children mostly develop a milder SARS-CoV-2 infection, while a reduced body temperature in older individuals favors a more severe course of the disease. Our data also have translational potential, as a temporary or rhythmic increase in core body temperature could be used as protective measure against viral infections.

## DATA AVAILABILITY

RNA-seq data is available at GSE193639. For reagents, please contact F.H.

## Supplementary Material

gkac513_Supplemental_Files
