# Supplementary material for: Body temperature variation controls pre-mRNA processing and transcription of antiviral genes and SARS-CoV-2 replication
Source: Nucleic Acids Res. 2022 Jun 17;50(12):6769–85. doi: 10.1093/nar/gkac513 (PMC9262603; doi:10.1093/nar/gkac513)
Supplement: gkac513_Supplemental_Files [file gkac513_supplemental_files.zip › Los_etal_SupplementaryMaterial.pdf]

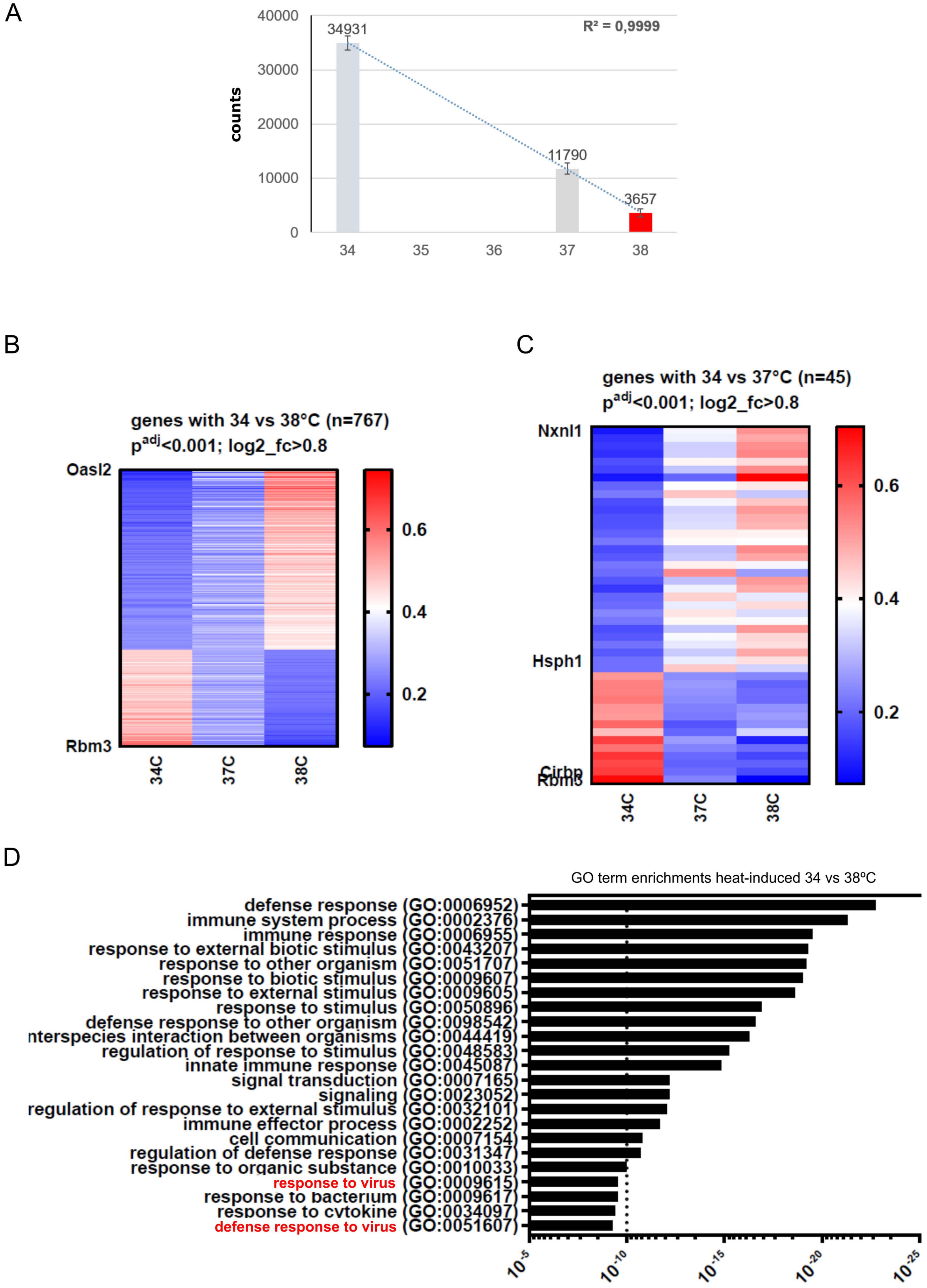

A
40000
30000
20000
10000
0
counts
34931
11790
3657
R² = 0,9999
34
35
36
37
38
B
genes with 34 vs 38°C (n=767)
p^adj<0.001; log2_fc>0.8
Oasl2
Rbm3
0.6
0.4
0.2
34C
37C
38C
C
genes with 34 vs 37°C (n=45)
p^adj<0.001; log2_fc>0.8
Nxnl1
Hsph1
Cirbp
Rbm3
0.6
0.4
0.2
34C
37C
38C
D
GO term enrichments heat-induced 34 vs 38ºC
defense response (GO:0006952)
immune system process (GO:0002376)
immune response (GO:0006955)
response to external biotic stimulus (GO:0043207)
response to other organism (GO:0051707)
response to biotic stimulus (GO:0009607)
response to external stimulus (GO:0009605)
response to stimulus (GO:0050896)
defense response to other organism (GO:0098542)
nterspecies interaction between organisms (GO:0044419)
regulation of response to stimulus (GO:0048583)
innate immune response (GO:0045087)
signal transduction (GO:0007165)
signaling (GO:0023052)
regulation of response to external stimulus (GO:0032101)
immune effector process (GO:0002252)
cell communication (GO:0007154)
regulation of defense response (GO:0031347)
response to organic substance (GO:0010033)
response to virus (GO:0009615)
response to bacterium (GO:0009617)
response to cytokine (GO:0034097)
defense response to virus (GO:0051607)
10^-5
10^-10
10^-15
10^-20
10^-25

Supp. Fig. 1. Changes in gene expression comparing 34°C and 37-38°C. A) Rbm3 is upregulated at lower temperatures in Raw264.7 cells. Expression levels of Rbm3 at different temperatures. The dotted line represents a linear regression fit of temperature and expression of Rbm3. **B)** Heatmap as in Fig. 1C showing significantly altered genes comparing 34°C and 38°C (n=767, sorted by change 34°C to 38°C). The 2′-5′ oligoadenylate synthetase like 2 (Oasl2) – important for viral RNA degradation – is the strongest heat-induced gene, while Rbm3 – a cold-induced RNA binding protein – is the strongest cold-induced gene. **C)** Heatmap as in Fig. 1C showing significantly altered genes comparing 34°C and 37°C (n=45, sorted by change 34°C to 37°. Cold-induced and warm-induced genes are highlighted on the left. GO term enrichments using these genes did not reveal any significant enrichments. **D)** Strongest GO term enrichments of 38°C-induced genes. GO terms containing the term ‘virus’ are highlighted in red. Note that these GO terms are less significantly enriched than in Fig. 1D, consistent with the notion that these genes are specifically induced by a one-degree increase in temperature from 37°C to 38°C. Cold-induced genes show no comparable significant GO term enrichments.

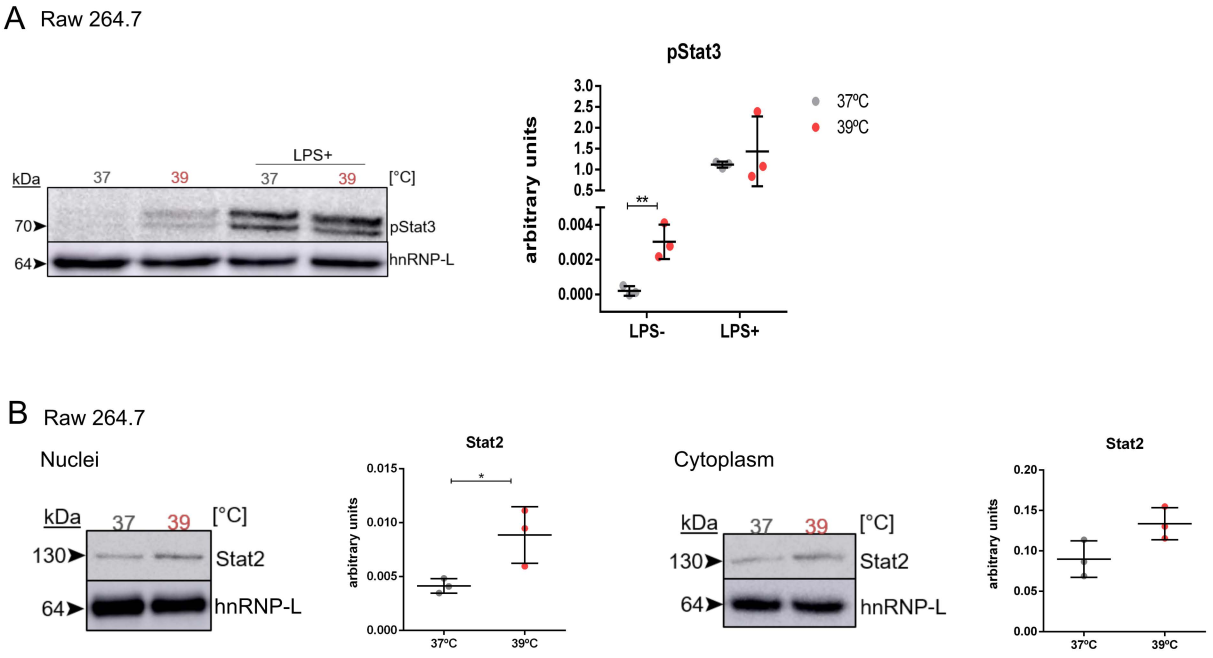

Supp. Fig. 2. Elevated temperature induces activation of STAT3 A) Raw264.7 cells were incubated at the indicated temperature for 12h and phosphorylation (activation) of STAT3 was analyzed by Western blot. As a positive control cells were incubated for 16h with LPS (2.25 µg/mL). A representative blot and quantification of three independent experiments are shown. hnRNP-L served as a loading control. **B)** Western blot showing expression of Stat2 in the nuclear and cytoplasmic fractions. A representative blot and quantification relative to hnRNP-L are shown (n=3).

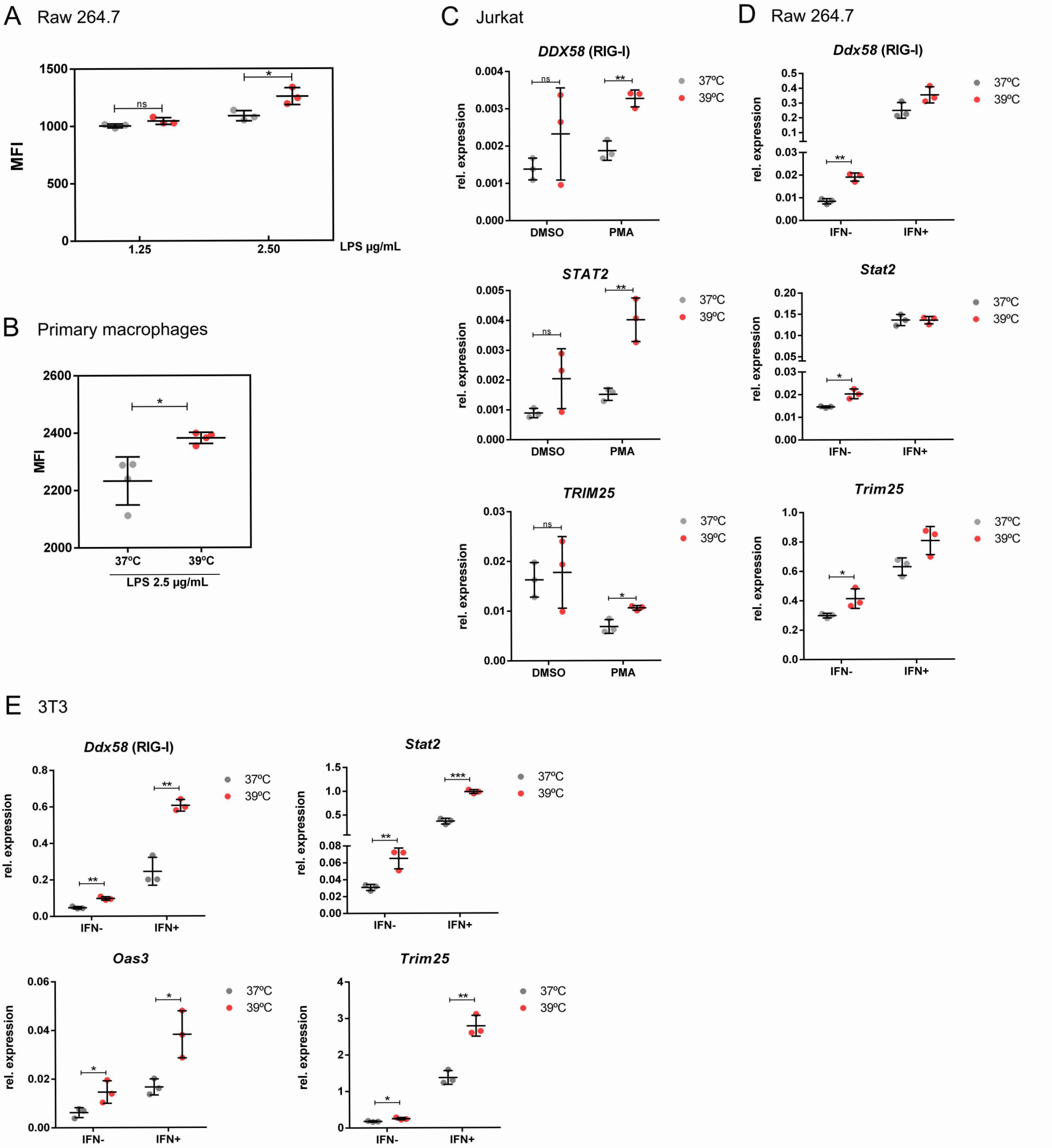

A Raw 264.7
MFI
LPS µg/mL
1.25
2.50
ns
*
B Primary macrophages
MFI
37ºC
39ºC
LPS 2.5 µg/mL
*
C Jurkat
DDX58 (RIG-I)
STAT2
TRIM25
rel. expression
DMSO
PMA
37ºC
39ºC
D Raw 264.7
Ddx58 (RIG-I)
Stat2
Trim25
rel. expression
IFN-
IFN+
37ºC
39ºC
E 3T3
Ddx58 (RIG-I)
Stat2
Oas3
Trim25
rel. expression
IFN-
IFN+
37ºC
39ºC

Supp. Fig. 3. Elevated temperature induces NO production in macrophages and upregulation of anti-viral genes in Jurkat cells and 3T3 cells. A) Quantification of mean fluorescence intensity of NO production from Fig. 3B. **B)** Same as in A) from Fig. 3D. **C)** Jurkat cells were incubated at different temperatures for 12h, then treated with 1ng/mL of phorbol myristate acetate (PMA) (or DMSO as control) and incubated at the indicated temperatures for an additional 16h. Expression of anti-viral genes was analyzed by RT-qPCR. mRNA expression is relative to *GAPDH*. (mean +/- SD, n=3). **D)** Raw264.7 cells were incubated for 12h at the indicated temperatures, and then 4h +/- IFN (100U/mL). Expression of anti-viral genes was analyzed by RT-qPCR. mRNA expression is relative to *Hprt*. (mean +/- SD, n=3). **E)** Increase expression of anti-viral genes at warmer temperatures in 3T3 cells. Cells were incubated and stimulated as in D). Expression of anti-viral genes was analyzed by RT-qPCR (mean +/- SD, n=3). Statistical significance was determined by unpaired t-tests and is indicated by asterisks, *p<0.05; **p<0.01; ***p<0.001; ns = not significant.

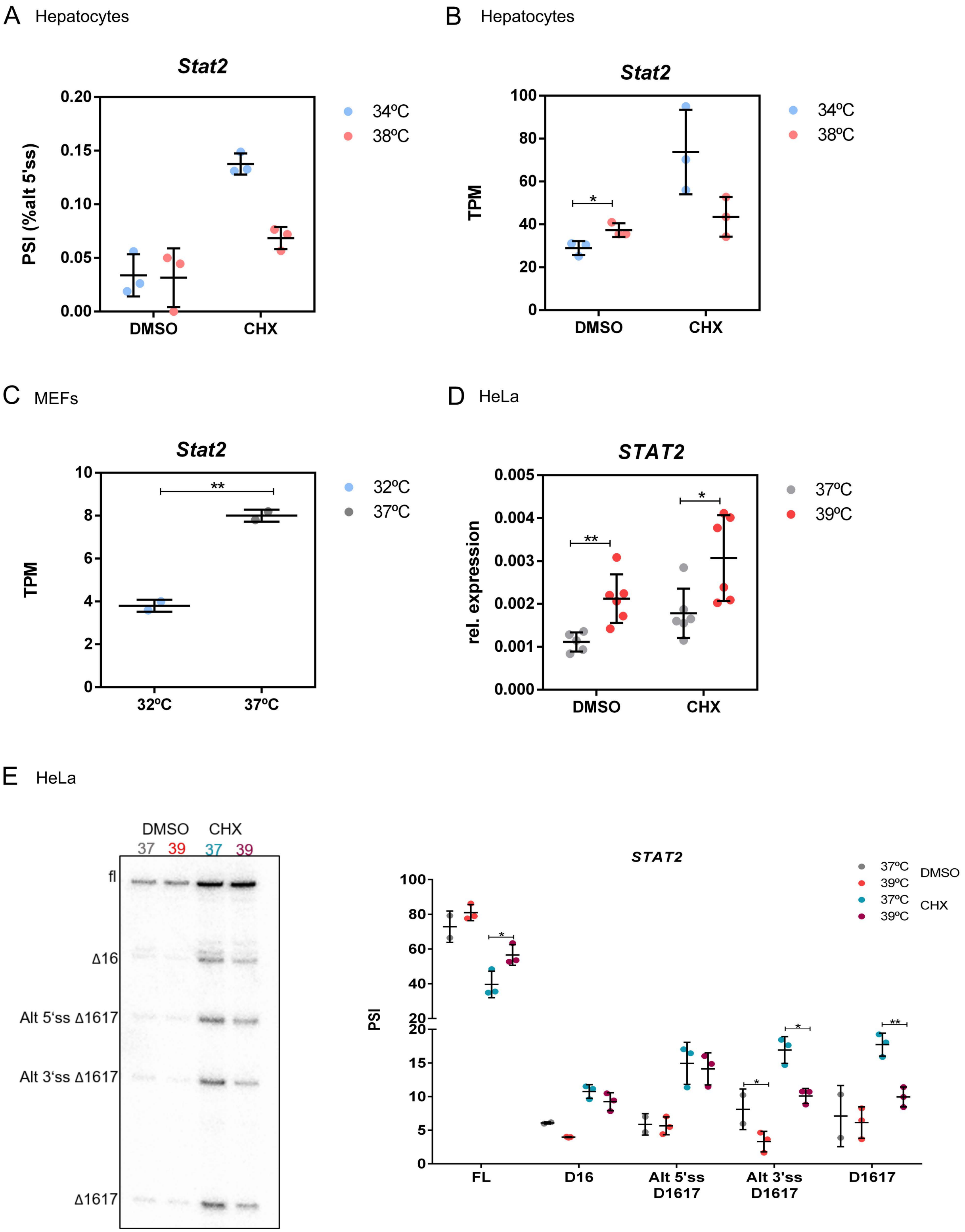
A Hepatocytes
Stat2
PSI (%alt 5'ss)
0.20
0.15
0.10
0.05
0.00
DMSO
CHX
34ºC
38ºC
B Hepatocytes
Stat2
TPM
100
80
60
40
20
0
DMSO
CHX
34ºC
38ºC
C MEFs
Stat2
TPM
10
8
6
4
2
0
32ºC
37ºC
32ºC
37ºC
D HeLa
STAT2
rel. expression
0.005
0.004
0.003
0.002
0.001
0.000
DMSO
CHX
37ºC
39ºC
E HeLa
DMSO
CHX
37
39
37
39
fl
Δ16
Alt 5'ss Δ1617
Alt 3'ss Δ1617
Δ1617
STAT2
PSI
37ºC
39ºC
DMSO
37ºC
39ºC
CHX
FL
D16
Alt 5'ss D1617
Alt 3'ss D1617
D1617

Supp. Fig. 4. *Stat2* alternative splicing coupled to nonsense-mediated decay in different cell lines. A) PSI values of the NMD-inducing 5' splice site in *Stat2* exon 11 shows reduced usage at 38°C in primary mouse hepatocytes treated with CHX (for details see (26). **B)** Expression levels of *Stat2* in transcript per million (TPM) in primary mouse hepatocytes in the absence (DMSO) or presence of CHX (see (26). **C)** Expression levels of *Stat2* in transcript per million (TPM) in mouse embryonic fibroblasts (MEFs) (data from (27). **D)** Expression levels of *STAT2* in HeLa cells. HeLa cells were incubated at the indicated temperature for 8h, then in the absence (DMSO) or presence of the CHX for an additional 4h. Gene expression was analyzed by RT-qPCR. mRNA expression is relative to *GAPDH* (n=6, mean +/- SD is shown). **E)** Radioactive, splicing-sensitive RT-PCR and phosphorimager quantification showing different *STAT2* NMD-induced isoforms in HeLa cells. Statistical significance was determined by unpaired t-tests and is indicated by asterisks, *p<0.05, **p<0.01.

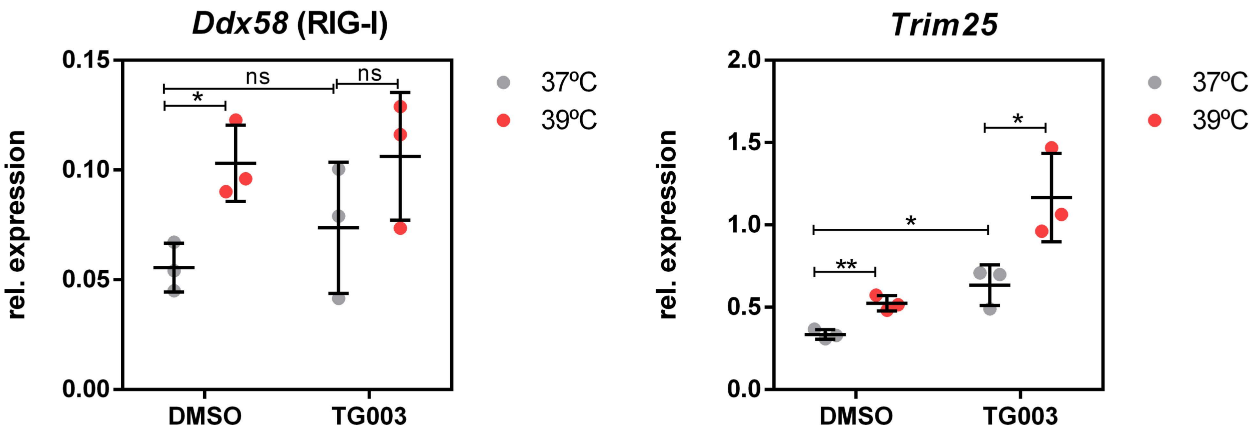

Supp. Fig. 5: Effect of CLK inhibition on the expression of anti-viral genes. Raw264.7 cells were incubated for 6h in the absence (DMSO) or presence of the CLK inhibitor TG003, then for an additional 12h at the indicated temperature. Expression of the indicated genes was analyzed by RT-qPCR (n=3, mean +/- SD is shown). Statistical significance was determined by unpaired t-tests and is indicated by asterisks, *p<0.05, **p<0.01, ns = not significant.

3T3

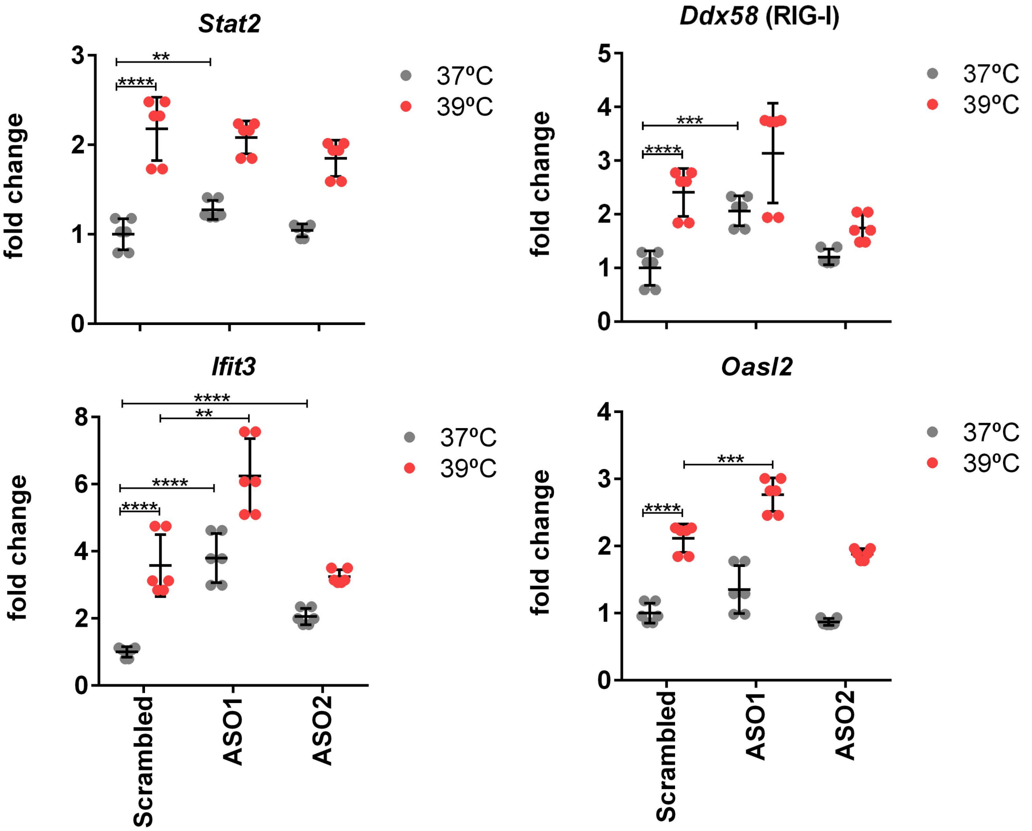

Supp. Fig. 6: Gene expression levels of the anti-viral genes upon ASO treatment in 3T3 cells. 3T3 cells were transfected with two different ASOs targeting the alternative 5' splice site in *Stat2* exon 11. 32h post-transfection, cells were incubated for 16h at 37°C or 39°C. Scrambled ASO was used as a control. Gene expression was investigated by RT-qPCR. mRNAs are normalized to scrambled ASO at 37°C (mean +/- SD, n=6). Statistical significance was determined by unpaired t-tests and is indicated by asterisks, *p<0.05; **p<0.01; ***p<0.001; ****p<0.0001.

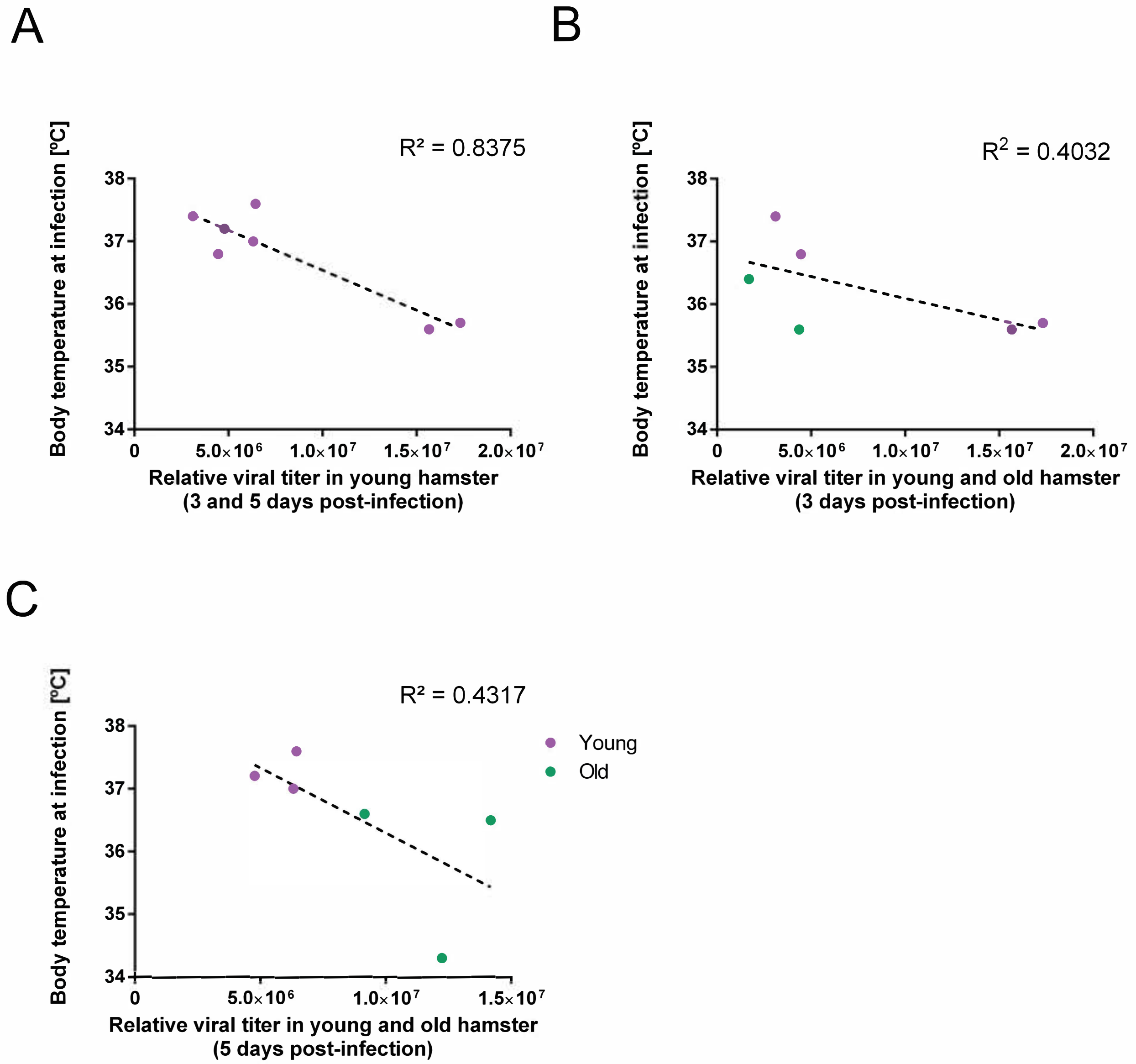

Supp. Fig. 7. SARS-CoV-2 replication is body temperature-controlled in Syrian hamsters. Published data from (31) was used to plot the body temperature of Syrian hamsters at the time point of SARS-CoV-2 infection against the viral mRNA detected in lungs 3 or 5 days post-infection. **A)** Young hamsters 3 and 5 days post-infection. **B)** Young and old hamsters 3 days post-infection. **C)** Young and old hamsters 5 days post-infection. The dotted line represents a linear regression fit of body temperature and viral mRNA.

Table S2. Primers sequence.

**Mice primers**	
mStat2_qF	GAAGATGAAGCTGCAGACGG
mStat2_intron_qF	TAACTCCGGTCTTCCTCCCAG
mStat2_qR	TTGGGCTGAGCATGTTGAAC
mDdx58_qF	CGACAAGGAAAACTGGCCAA
mDdx58_intron_qF	AGAAACCCTGCCTCGACCAC
mDdx58_qR	CTCCGCTCCATCATCCTCAT
mTrim25_qF	CGGAAAATTCGACACCATCT
mTrim25_intron_qF	CCCTGGCTTTCCTCCAATCAC
mTrim25_qR	CTTAGGGATGTAGACTGGTT
mMavs_qF	AGAGCAACTCCTCCAGACCA
mMavs_qR	AACGGTTGGAGACACAGGTC
mIrf7_qF	TGATCCGCATAAGGTGTACG
mIrf7_qR	GCATCACTAGAAAGCAGAGG
mIfit3_qF	CAGCAGCACAGAAACAGATC
mIfit3_qR	CTGGACATACTTCCTTCCCT
mOasl2_qF	TAAAGGTTCAGTCCCGGAAG
mOasl2_qR	CAGGGTAGCCCTTACTTCTT
mOas3_qF	TAATCCTGCTGGTCAAACAC
mOas3_qR	GGTACTTGCTGTGTTGGATC
mNos2_qF	GGAATCTTGGAGCGAGTTGT
mNos2_qR	CCTCTTGTCTTTGACCCAGTAG
mHprt_qF	CAACGGGGGACATAAAAGTTATTGGTGGA
mHprt_qR	TGCAACCTTAACCATTTTGGGGCTGT
mSTAT2_E11_F	CTGGTGAGACTCCAGGAAGG
mSTAT2_E13_R	AAGAAGCCGAAGTCCCAAAT
**Human primers**	
hStat2_qF2	GAAGCAGGAGCTGAAAACGG

hStat2_qR2	TTGGGCTGAGCAAATTGAAC
hDdx58_qF	GCGTCAGTGATAGCAACAGTC
hDdx58_qR	GTCGCTAATCCGTGATTCCAC
hTrim25_qF	GAGGAGATTGAACAGAGCCTGAC
hTrim25_qR	CTGGCTTTGTTGAGATTCCTCG
hOas1_qF	GAGAAGGCAGCTCACGAAAC
hOas1_qR	CCTCCAACCCTTTGGGTCTC
hGapdh_qF	CTTCGCTCTCTGCTCCTCCTGTTCG
hGapdh_qR	ACCAGGCGCCCAATACGACCAAAT
hSTAT2_E15_fwd	TGCACATCATCAGCTTCACG
hSTAT2_E18_rev	TTAGTGAAGTCAGCCCAGGA
