# Supplementary material for: Body temperature variation controls pre-mRNA processing and transcription of antiviral genes and SARS-CoV-2 replication
Source: Nucleic Acids Res. 2022 Jun 17;50(12):6769–85. doi: 10.1093/nar/gkac513 (PMC9262603; doi:10.1093/nar/gkac513)
Supplement: gkac513_Supplemental_Files [file gkac513_supplemental_files.zip › TableS1.docx]

**Table S1.** Temperature-dependent changes in gene expression in Raw264.7 cells. RNA-seq data related to Figure 1.

**Sheet 1:** Differentially expressed genes between 37°C and 38°C.

**Sheet2.** Differentially expressed genes between 34°C and 37°C.

**Sheet3.** Differentially expressed genes between 34°C and 38°C.

**Sheet4.** All expressed genes associated with the GO term ‘defense response to virus’ and their temperature-dependent expression.

Genes were considered significantly changed with a log2 fold change > 0.8 (< -0.8) and a p^adj^ < 0.001 between two temperatures.
